# Social and novel contexts modify hippocampal CA2 representations of space

**DOI:** 10.1038/ncomms10300

**Published:** 2016-01-25

**Authors:** Georgia M. Alexander, Shannon Farris, Jason R. Pirone, Chenguang Zheng, Laura L. Colgin, Serena M. Dudek

**Affiliations:** 1 Neurobiology Laboratory, National Institute of Environmental Health Sciences, National Institutes of Health, 111 T.W. Alexander Drive, Mail Drop F2-04, Research Triangle Park, North Carolina 27709, USA; 2 Social and Scientific Systems, Inc., 1009 Slater Road Suite 120, Durham, North Carolina 27703, USA; 3 Center for Learning and Memory, The University of Texas at Austin, 1 University Station Stop C7000, NMS 4.104, Austin, Texas 78712, USA; 4 Department of Neuroscience, The University of Texas at Austin, Austin, Texas 78712, USA

## Abstract

The hippocampus supports a cognitive map of space and is critical for encoding declarative memory (who, what, when and where). Recent studies have implicated hippocampal subfield CA2 in social and contextual memory but how it does so remains unknown. Here we find that in adult male rats, presentation of a social stimulus (novel or familiar rat) or a novel object induces global remapping of place fields in CA2 with no effect on neuronal firing rate or immediate early gene expression. This remapping did not occur in CA1, suggesting this effect is specific for CA2. Thus, modification of existing spatial representations might be a potential mechanism by which CA2 encodes social and novel contextual information.

## Abstract (web summary)

Recent work has implicated hippocampal subfield CA2 in encoding social and contextual memory yet the neural mechanisms are not known. Here, Alexander and colleagues demonstrate that, compared to CA1 neurons, CA2 neurons modify their place fields when presented with social or novel stimuli.

## Graphical abstract

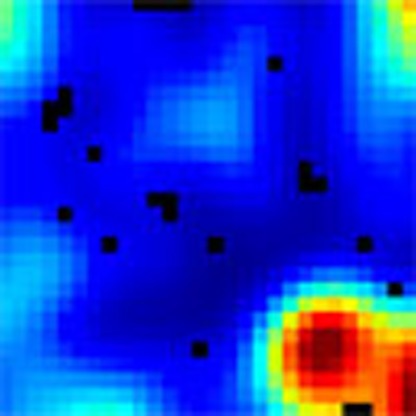

Hippocampal area CA2 has become increasingly appreciated as a distinct region based on several unique synaptic^1^,^2^, molecular^3^ and anatomical^4^ properties that suggest specific roles for CA2 in hippocampal function^5^. Recently, behavioural studies in both rats and mice have implicated CA2 in spatial, contextual and social memory^6^,^7^,^8^,^9^,^10^,^11^,^12^. Neurons in CA1 and CA3 preferentially fire in distinct regions of a two-dimensional spatial environment, called place fields^13^,^14^,^15^. CA2 neurons in rats were recently also found to fire in place fields, although CA2 place cells encoded less spatial information than CA1 and CA3 place cells^6^,^7^. Furthermore, in contrast to CA1 and CA3, at the population level, CA2 ensembles remapped to a greater degree over the passage of time (hours to days) than in response to changing the shape of a familiar context, suggesting that CA2 is less sensitive to spatial and contextual information than CA1 or CA3. Other recent studies have found that CA2 ensembles remap to novel contexts as measured by *in vivo* electrophysiology in rats^7^ and cellular compartment analysis of temporal activity by fluorescent *in situ* hybridization (catFISH) in mice^9^. Indeed, CA2 was found to be the most sensitive CA region to remapping when a familiar context was updated with novel objects^9^. Therefore, whether CA2 neurons encode novel versus contextual information is unclear and warrants further investigation.

A role for CA2 in social behaviour had been predicted based on the findings that expression of the vasopressin 1b receptor (*Avpr1b*), a receptor for the social neuropeptide vasopressin, is highly restricted in the brain to CA2 neurons^16^, and *Avpr1b*-null mice show deficits in social recognition memory and social aggression^17^,^18^. Further, restoration of *Avpr1b* expression in dorsal CA2 of *Avpr1b*-null mice was sufficient to rescue social aggression behaviours^10^. In addition, silencing CA2 neuronal output with tetanus toxin light chain results in a selective loss of social recognition memory with no impairment in other hippocampal-dependent memory tasks^11^. Whether and how CA2 neurons encode this form of social information is unknown.

In this study, we use *in vivo* single neuron recording and immediate early gene (IEG) mapping in male rats to determine how CA2 neurons respond to social and contextual experiences. We present evidence that CA2 place fields globally remap in response to social and novel contextual experiences. Interestingly, firing rates and IEG expression were unaffected by social experience. Thus, CA2 neurons have the capacity to update their spatial representations online in response to social or contextual changes in the environment, possibly as a means to encode social and novel contextual information. These results suggest that CA2 place cells may link social and novel contextual information with representations of space.

## Results

### CA2 encodes spatial information

**Figure 1:**
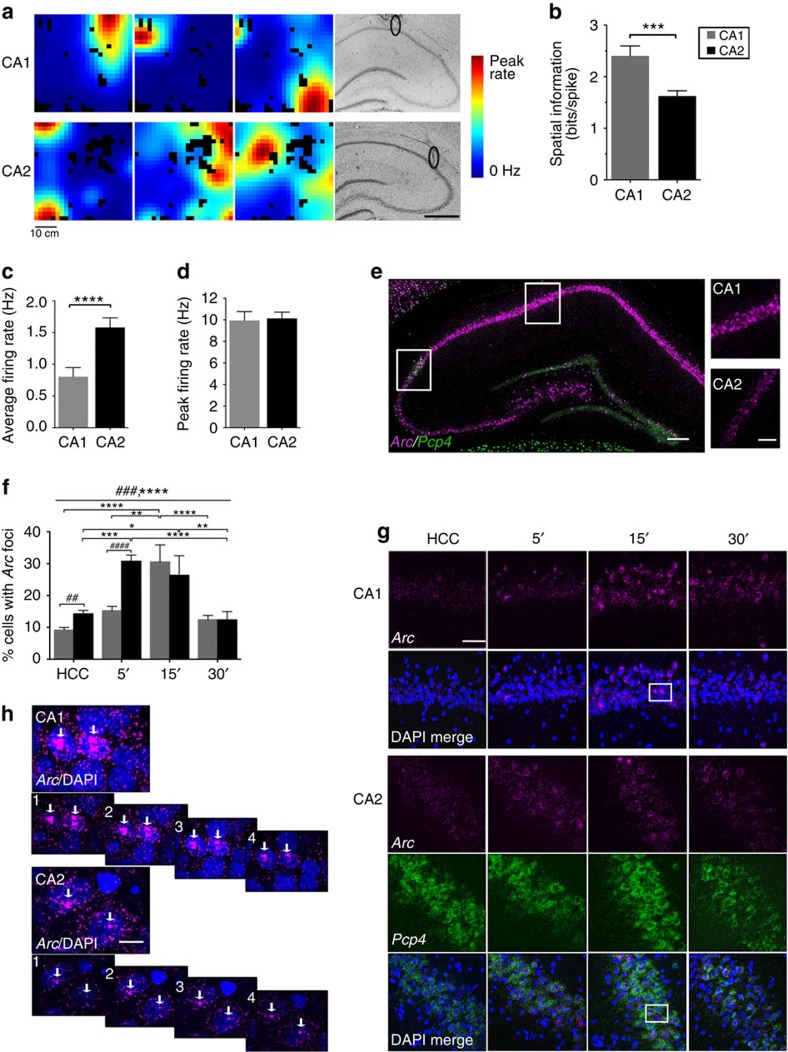
CA2 encodes spatial information. (**a**) Place field maps for three neurons and electrode tracks for CA1 and CA2. (**b**) Spatial information calculated for CA1 and CA2 neurons. CA2 neurons carried less spatial information than CA1 neurons (median(CA1)=2.191, median(CA2)=1.536, *U*=741, *P*<0.001, Mann–Whitney test, *n*=61 CA2 neurons and 31 CA1 neurons). (**c**) CA2 place cells had a higher average firing rate than CA1 place cells (median (CA1)=0.5146, median(CA2)=1.354, *U*=468, *P*<0.0001, Mann–Whitney test, *n*=61 CA2 neurons and 31 CA1 neurons). (**d**) CA1 and CA2 place cells had similar peak firing rates (median(CA1)=9, median(CA2)=9, *U*=860, *P*>0.05, Mann–Whitney test, *n*=61 CA2 neurons and 31 CA1 neurons). (**e**) Representative smFISH probing for *Arc* and *Pcp4* in dorsal hippocampus from a rat that was killed 30 min after a 10-min exploration. (**f**) Time course of the percentage of cells with *Arc* foci in CA1 and CA2 following spatial exploration (main effect of time F(3,25)=11.09, *P*<0.0001; main effect of subregion F(1,25)=19.47, *P*<0.001, two-way paired ANOVA with Bonferroni *post hoc* tests denoted on graph; *n*=5–9 rats per condition). (**g**) Representative images of data in **f**. (**h**) High-magnification images of neurons outlined by white boxes in **g**. Arrows denote *Arc* foci throughout *z*-plane. HCC, home cage controls. Scale bar, 500 μm (**a**), 200 μm (**e**, left image), 50 μm (**e**, right image and **g**), 10 μm (**h**). *P<0.05; **P<0.01; ***P<0.001, ****P<0.0001. Error bars are s.e.m.

First, we assessed the spatial properties of CA2 and CA1 neurons using single unit recordings from awake, behaving adult male rats, while animals explored a familiar open field arena (Supplementary Fig. 1). Spatial properties were assessed while the animal actively explored. We included data from periods of locomotion and active whisking, but not quiet wakefulness, or brief periods of immobility. Consistent with previous studies^6^,^7^, CA2 neurons fired in place fields but differed from CA1 neurons in that they had larger place fields (*P*=0.0019, one-tailed unpaired Mann–Whitney test, *n*=61 CA2, 31 CA1 neurons; Supplementary Fig. 2c) and higher average firing rates during exploration (*P*<0.0001, one-tailed unpaired Mann–Whitney test, *n*=61 CA2, 31 CA1 neurons; Fig. 1c). Thus, CA2 neurons carried less spatial information than neurons recorded in CA1 (*P*<0.001, one-tailed unpaired Mann–Whitney test, *n*=61 CA2, 31 CA1 neurons; Fig. 1a,b). Although the average firing rate was higher in CA2, the peak firing rate was not different from CA1 (*P*>0.05, one-tailed unpaired Mann–Whitney test, *n*=61 CA2, 31 CA1 neurons; Fig. 1d). When compared with quiet wake, a behavioural state distinct from sleep, CA2 and CA1 neurons both had a higher average firing rate during exploration (CA2: *P*<0.001; CA1: *P*<0.001, *n*=61 CA2, 31 CA1 neurons, Wilcoxon signed-rank test; Supplementary Fig. 3), as well as a higher peak firing rate (CA2: *P*<0.0001; CA1: *P*<0.0001, *n*=61 CA2, 31 CA1 neurons, Wilcoxon signed-rank test; Supplementary Fig. 3). Thus, both populations of neurons were more active during exploration than quiet wake.

To gain a better understanding of CA2 neuronal activity during spatial exploration at the population level, we measured IEG expression at different time points after the onset of exploration (5, 15 and 30 min). Separate cohorts of adult male rats explored a familiar open field arena for either 3 or 10 min and were returned to their home cage for the remaining time until they were killed at 5 min (for the 3-min exploration condition), or 15 or 30 min (for the 10-min exploration conditions). Multiplexed FISH was used to probe for *activity-regulated cytoskeleton-associated protein* (*Arc*)*, early growth response 1* (*Egr1*, also known as *Zif268*) and *purkinje cell protein 4* (*Pcp4*) within the same sections. CA2 was defined by detection of *Pcp4*, the expression of which delineates the CA1/CA2 and CA2/CA3 borders (Fig. 1e). We also verified immunostaining for striatal-enriched protein phosphatase (STEP, also known as PTPN5) and regulator of G-protein signalling 14, which were used previously to delineate mouse CA2 (Supplementary Fig. 4)^3^,^4^,^19^. IEG mapping revealed an increased percentage of CA2 neurons that were positive for *Arc* transcription foci at 5 and 15 min, which returned to baseline by 30 min (main effect of time, *P*<0.0001, main effect of subregion *P*=0.0002, two-way analysis of variance (ANOVA), *n*=5–10 rats per condition; Fig. 1f,g). CA1 neurons showed peak *Arc* transcription at 15 min, also returning to baseline by 30 min, similar to previous findings^20^,^21^. The percentage of neurons with *Egr1* transcription foci was significantly increased in CA1 at 5, 15 and 30 min (CA1: main effect of time *P*<0.0001, main effect of group *P*<0.0001, two-way paired ANOVA, *n*=5–10 rats per condition; Supplementary Fig. 5a), consistent with previous findings^22^. In CA2, the increase in percentage of neurons with *Egr1* foci was significant only at the 5-min time point (*P*<0.0001 Bonferroni *post hoc* test; two-way paired ANOVA, main effect of time *P*<0.0001, main effect of group *P*<0.0001, *n*=5–10 rats per condition; Supplementary Fig. 5b). Increased expression of IEGs after spatial exploration typically indicates that neurons were activated during the behaviour. Notably though, IEG transcription foci in CA2 were considerably smaller than those in CA1 (Fig. 1h), consistent with the overall lower levels of *Arc* messenger RNA found in CA2 (Fig.1e).

### CA2 activity levels are not modified by social stimuli

**Figure 2:**
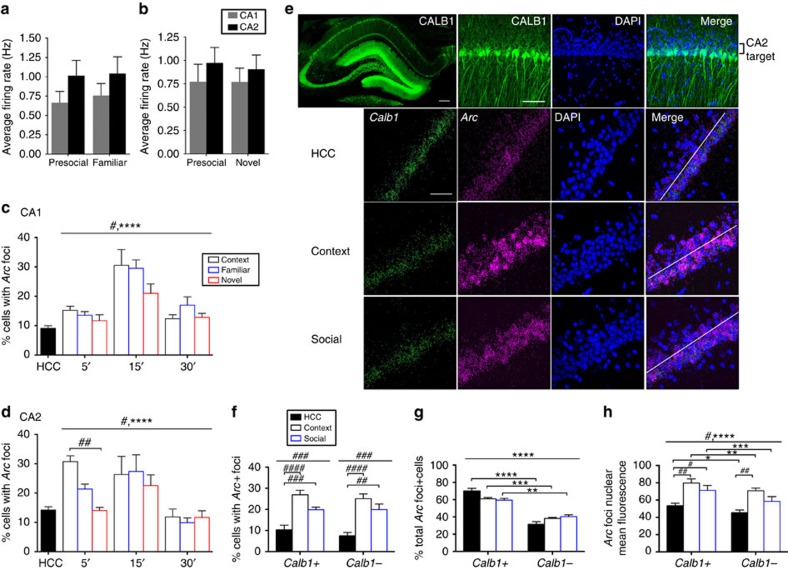
CA2 activity levels are not modified by social stimuli. (**a**,**b**) Average firing rate before and during exposure to social stimuli for CA1 and CA2. No differences in average firing rates were detected for CA2 neurons during familiar exposure (median(pre-social)=0.5850, median(familiar)=0.5271, *W*=43.0, *P*>0.05, *n*=49 CA2 neurons) or novel exposure (median(pre-social)=0.5278, median(familiar)=0.5280, *W*=−162.0, *P*>0.05, *n*=55 CA2 neurons, Wilcoxon signed-rank test) or for CA1 neurons during familiar exposure (median(pre-social)=0.3210, median(familiar)=0.3800, *W*=336.0, *P*>0.05, *n*=55 CA1 neurons) or novel exposure (median(pre-social)=0.2875, median(familiar)=0.3682, *W*=431.0, *P*>0.05, *n*=58 CA1 neurons, Wilcoxon signed-rank test). (**c**,**d**) Time course of percentage of cells with *Arc* foci in CA1 and CA2 following spatial and social exploration (CA1: main effect of time F(2,48)=25.13, *P*<0.0001; main effect of condition F(2,48)=3.198, *P*=0.0497, two-way ANOVA. Bonferroni *post hoc* tests revealed no significant difference between conditions at any time point; however, context versus novel at 15 min trended towards significance (*P*=0.055). CA2: main effect of time F(2,48)=18.72, *P*<0.0001; main effect of condition F(2,48)=3.400, *P*=0.0416, two-way ANOVA. Bonferroni *post hoc* results are shown for the main effect of condition. See Supplementary Table 1 for *post hoc* tests for the main effect of time. HCC conditions are shown for comparison but were not included in the statistics. *n*=5–9 rats per condition). (**e**) Immunostaining of calbindin-1 (CALB1) in dorsal hippocampus and CA1 to delineate the target layer of CA2 axons and representative smFISH images probing for *Calb1* and *Arc* across conditions. (**f**) Percentage of *Calb1+* or *−* cells expressing *Arc* foci across conditions (main effect of condition F(2,13)=19.59, *P*=0.0001, two-way paired ANOVA with Bonferroni *post hoc* tests; *n*=5–6 rats per condition). (**g**) Per cent total *Arc* foci+cells that are *Calb1+* or *Calb1−* (main effect of *Calb1* expression F(1,13)=107.8, *P*<0.0001, two-way paired ANOVA with Bonferroni *post hoc* tests). (**h**) *Arc* mean nuclear fluorescence per cell measured in *Arc* foci+cells as a function of *Calb1* expression (main effect of *Calb1* expression F(1,13)=56.94, *P*<0.0001; main effect of condition F(2,13)=8.155, *P*=0.0051, two-way paired ANOVA with Bonferroni *post hoc* tests). Scale bar, 200 and 50 μm (left and right/lower). #,*P<0.05; ##,**P<0.001; ###,***P<0.001; ####,****P<0.0001. Error bars are s.e.m.

Because of the suggested role of CA2 in social behaviour, we next asked whether CA2 activity is modified by social stimuli. In separate cohorts of rats we measured average firing rate and IEG expression on addition of a familiar or novel rat into a cage insert within the open-field arena. The insert permitted olfactory, visual and limited tactile interaction with the conspecific through holes on the bordering wall of the insert (Supplementary Fig. 1c). Novel animals were housed in separate colony rooms and familiar animals were previous cage mates housed in the same colony room as the subject animal. The social exposure was the first time the rats encountered each other in this arena. Social stimulation did not cause an increase in average firing rate of CA2 neurons over levels induced by exploration alone (*P*>0.05 for novel and familiar, Wilcoxon matched-pairs signed-rank test; Fig. 2a,b, *n*=55 neurons for novel, *n*=49 neurons for familiar; see Supplementary Fig. 6 for analyses including CA2 place cells only). Similarly, the percentage of CA2 neurons expressing *Arc* foci after social exploration (familiar and novel) at the peak time point (15 min) was similar to the percentage after exploration of the context alone (*P*>0.05 Bonferroni *post hoc* tests across conditions at 15 min, two-way ANOVA, main effect of time *P*<0.0001, main effect of condition *P*=0.0416, *n*=5–9 rats per condition; Fig. 2d). The percentage of CA1 neurons expressing *Arc* foci after familiar social exploration at the peak time point (15 min) was also similar to the percentage after exploration alone (*P*>0.05 Bonferroni *post hoc* tests across conditions at 15 min, two-way ANOVA, main effect of time *P*<0.0001, main effect of condition *P*=0.0497, *n*=5–9 rats per condition; Fig. 2c). These data indicate that social interaction caused no further increase in CA2 activity over exploration alone. We also investigated whether other survival-relevant olfactory cues (predator or prey scents) could activate CA2 neurons independent of exploration. We found that *Arc* expression was not significantly different following these exposures (Supplementary Fig. 7 and Supplementary Methods).

As CA2 activity levels did not change with social exposure, we hypothesized that CA1, the output of CA2, could have integrated a change in input weights from CA2, CA3 and ECIII on social exposure to selectively shift the population of active CA1 neurons to those targeted by CA2. We therefore assessed the possibility that social stimuli may have had a more robust effect on activity of CA2 target neurons (the deep CA1 neurons) than we could detect in CA2. Kohara *et al*.^4^ reported that the predominant target of CA2 axon terminals are the calbindin-1 negative (CALB1−) neurons in the deep layer of CA1 stratum pyramidale (Fig. 2e), presenting an opportunity to study putative CA2 target neurons in CA1. To examine whether social stimuli modify activity of this population of CA1 neurons, we measured the percentage of *Calb1*+ or *Calb1−* neurons expressing *Arc* foci and the mean nuclear *Arc* fluorescence of *Arc* foci+ neurons at the 15-min time point, when the percentage of neurons expressing *Arc* foci was greatest (Fig. 2c and Supplementary Fig. 8). Across all conditions (home cage control (HCC), context and social), a similar percentage of *Arc+* foci were detected in the *Calb1*+ and *Calb1*− populations (main effect of condition *P*<0.0001, two-way paired ANOVA, *n*=5–6 rats per condition; Fig. 2f). However, the *Calb1*+ population made up ∼60% of total CA1 cells (*Calb1+*: 60.21% s.d. 1.639; *Calb1−*: 39.79% s.d. 1.639); thus, about 60% of *Arc+* neurons were *Calb1+* (main effect of *Calb1* expression *P*<0.0001, two-way paired ANOVA, *n*=5–6 rats per condition; Fig. 2g). Despite the fact that the percentage of cells with *Arc* foci was similar in the *Calb1+* and *Calb1−* populations across conditions (Fig. 2f), the average amount of nuclear *Arc* expression in *Arc* foci+ neurons was consistently lower in *Calb1−* neurons than *Calb1*+ neurons for all conditions (*P*<0.0001 main effect of *Calb1* expression, *P*<0.001 main effect of condition, two-way paired ANOVA, *n*=5–6 rats per condition; Fig. 2h). These data suggest that the *Calb1−* neurons might be less active than the *Calb1*+ neurons, and that although both populations had increased *Arc* expression after spatial exploration, the distribution of *Arc*-expressing neurons was not greater in the *Calb1−* population after social stimuli.

### CA2 firing globally remaps on social and novel stimuli

**Figure 3:**
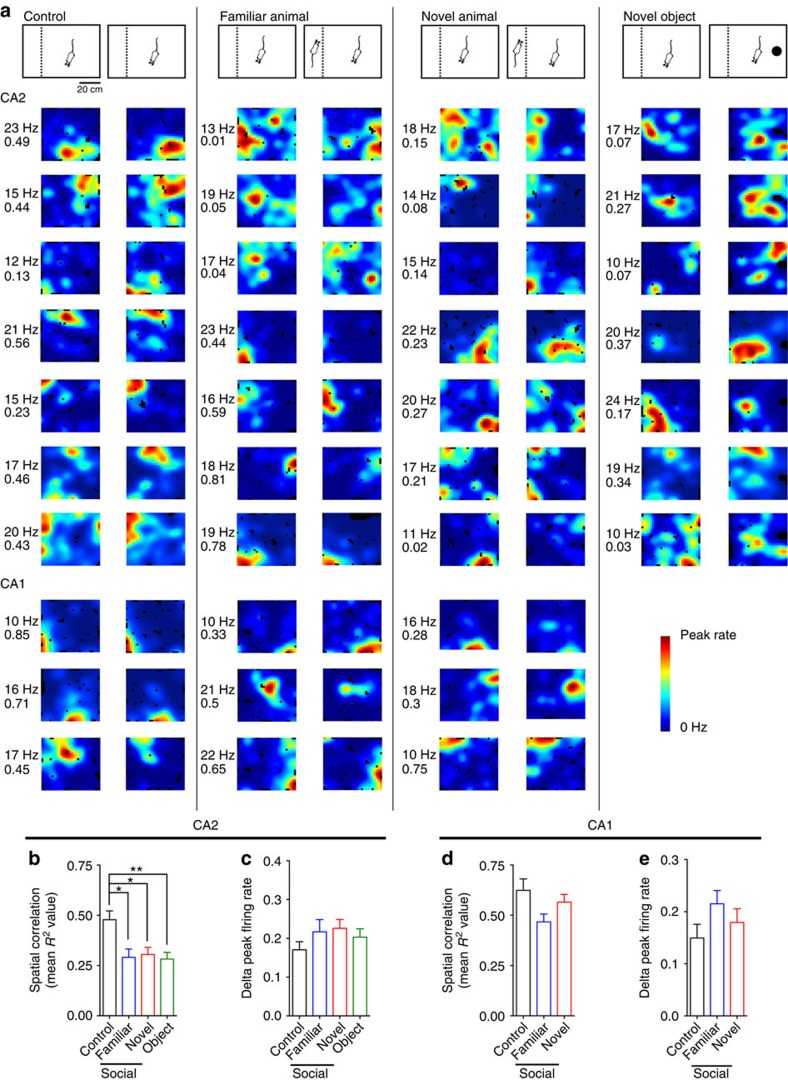
CA2 globally remaps on exposure to social and contextual stimuli. (**a**) Example place maps for CA2 neurons (top) and CA1 neurons (bottom) on exposure to each of the four conditions depicted in the diagrams on top. In the first epoch of each of the four conditions, animals explored the open-field arena with no stimulus present. In the second epoch, animals were exposed to no stimulus (control), a familiar rat, a novel rat or a novel object. Stimulus rats were placed in a clear plexiglass chamber and novel objects were placed inside the open-field arena. For each place field map pair, the peak firing rate for the pair (in Hz, top) and the spatial correlation value (bottom) is listed to the left and heat maps for both place field maps are scaled to the peak rate. (**b**,**c**) Analyses of remapping of firing fields by CA2 neurons on the four exposures listed, including spatial correlations to measure global remapping (**b**) and changes in peak firing rates (**c**). CA2 place cells globally remapped their representation of space in response to familiar and novel rats and novel objects compared with control conditions with no social exposure (H(3,168)=13.32, *P*<0.01, Kruskal–Wallis test with Holm–Bonferroni correction; Fig. 3a,b, *n*=31, 37, 54 and 46 CA2 neurons for control, familiar social, novel social and novel object exposures). Global remapping occurred in CA2 without any significant changes in peak firing rate (*H*(3,168)=1.649, *P*>0.05, Kruskal–Wallis test, same *n* values as **b**). Spatial correlation values (**d**) and changes in peak firing rates (**e**) for CA1 neurons, which showed no global remapping (*H*(2,144)=5.434, *P*=0.0661, Kruskal–Wallis test, *n*=28, 59 and 57 neurons for control, familiar social and novel social, respectively) or changes in peak firing rate (*H*(2,144)=2.895, *P*>0.05, Kruskal–Wallis test, same *n* values as **d**) during control or social stimulation. *P<0.05; **P<0.01. Error bars are s.e.m.

Given that we were unable to detect changes in CA1 or CA2 activity levels during social stimulation, we considered the possibility that non-spatial information such as social stimuli is encoded by modifications in representations of space (place fields) in CA2. To test this possibility, we monitored activity of individual CA2 and CA1 neurons *in vivo* before and during exposure to (1) a familiar male rat (prior cage mate), (2) a novel male rat or (3) a control condition with no social exposure to control for the passage of time^6^. We then compared the resulting place maps before and during the exposures by calculating spatial correlation values, which measure the extent to which place maps change from one condition to another. Surprisingly, we found that CA2 place maps were significantly changed in response to both familiar and novel rat exposure compared with control conditions (*P*<0.01, Kruskal–Wallis test with Holm–Bonferroni correction, control: *n*=31 neurons, familiar social: *n*=37, novel social: *n*=54; Fig. 3a,b). Global remapping occurred in CA2 without any significant changes in peak firing rate at the population level (*P*>0.05, Kruskal–Wallis test, control: *n*=31 neurons, familiar social: *n*=37, novel social: *n*=54; Fig. 3c). Consistent with recent reports^6^,^7^, the mean spatial correlation between control sessions was lower for CA2 than for CA1 (*P*<0.01 unpaired Mann–Whitney test, CA2: *n*=31 neurons, CA1: *n*=28; Fig. 3b,d and Supplementary Fig. 9).

As CA1 is the primary target of CA2 neurons, we expected some effect of social stimuli on CA1 spatial representations. CA1 spatial correlation values across the three conditions (control, familiar animal and novel animal exposures) were not significantly different from each other but did show a trend in that direction (*P*=0.0661, Kruskal–Wallis test, control: *n*=28 neurons, familiar social: *n*=59, novel social: *n*=57; Fig. 3a,d). We also found no significant changes in peak firing rate in CA1 at the population level on presentation of social stimuli (*P*>0.05, Kruskal–Wallis test, control: *n*=28 neurons, familiar social: *n*=59, novel social: *n*=57; Fig. 3e).

Comparing CA2 and CA1 spatial correlations across the three conditions showed a main effect of brain area (*P*<0.0001, F(1, 260)=39.49, two-way ANOVA, control: *n*=31 CA2 and 28 CA1 neurons, familiar social: *n*=37 CA2 and 59 CA1 neurons, novel social: *n*=54 CA2 and 57 CA1 neurons) and a main effect of exposure (*P*<0.01, F(1, 260)=6.512) but no significant interaction (statistical results not labelled on Fig. 3). Bonferroni *post hoc* tests showed significant differences between CA2 and CA1 spatial correlation values on familiar (*P*<0.01) and novel animal (*P*<0.0001) exposure but not during control exposure (*P*>0.05).

To determine whether the remapping in CA2 was specific to social exposure, we also measured spatial correlations between maps before and during rats' exposure to a novel object. Similar to social stimuli, exposure to a novel object significantly changed CA2 place maps (*P*<0.01, Kruskal–Wallis test with Holm–Bonferroni correction, control: *n*=31 neurons, novel object: *n*=46; Fig. 3a,b) without changing peak firing rates (*P*>0.05, Kruskal–Wallis test, control: *n*=31 neurons, novel object: *n*=46; Fig. 3c). Finally, we assessed whether CA2 firing patterns could differentiate among stimuli (familiar or novel rat) by measuring the direction of place field shifts on presentation of the stimuli. Using a centre of mass analysis (see Methods), we did not detect a bias in the direction of place field shifts for any condition (*P*>0.05 for control, familiar animal and novel animal, Rayleigh test, control: *n*=31 neurons, familiar social: *n*=37 neurons, novel social: *n*=54 neurons; Supplementary Fig. 12).

Although we did not detect a change in peak firing rate in CA1 or CA2 at the population level, assessment of individual neurons revealed a small number of CA2 and CA1 neurons that showed changes in peak firing rate of >50% (7 and 10 neurons, respectively, across all conditions; Supplementary Fig. 10). Comparing spatial correlation values in the absence of these neurons revealed similar results; CA2 neurons showed significant decreases in spatial correlations for novel and familiar animal conditions (*P*<0.01, Kruskal–Wallis test with Holm–Bonferroni correction, control: *n*=31 neurons, familiar animal: *n*=34 neurons, novel animal: *n*=51 neurons, novel object: *n*=45 neurons), but CA1 neurons showed no significant changes in spatial correlation values for social conditions (*P*>0.05, Kruskal–Wallis test, control: *n*=28 neurons, familiar animal: *n*=53 neurons, novel animal: *n*=53 neurons; Supplementary Fig. 10).

**Figure 4:**
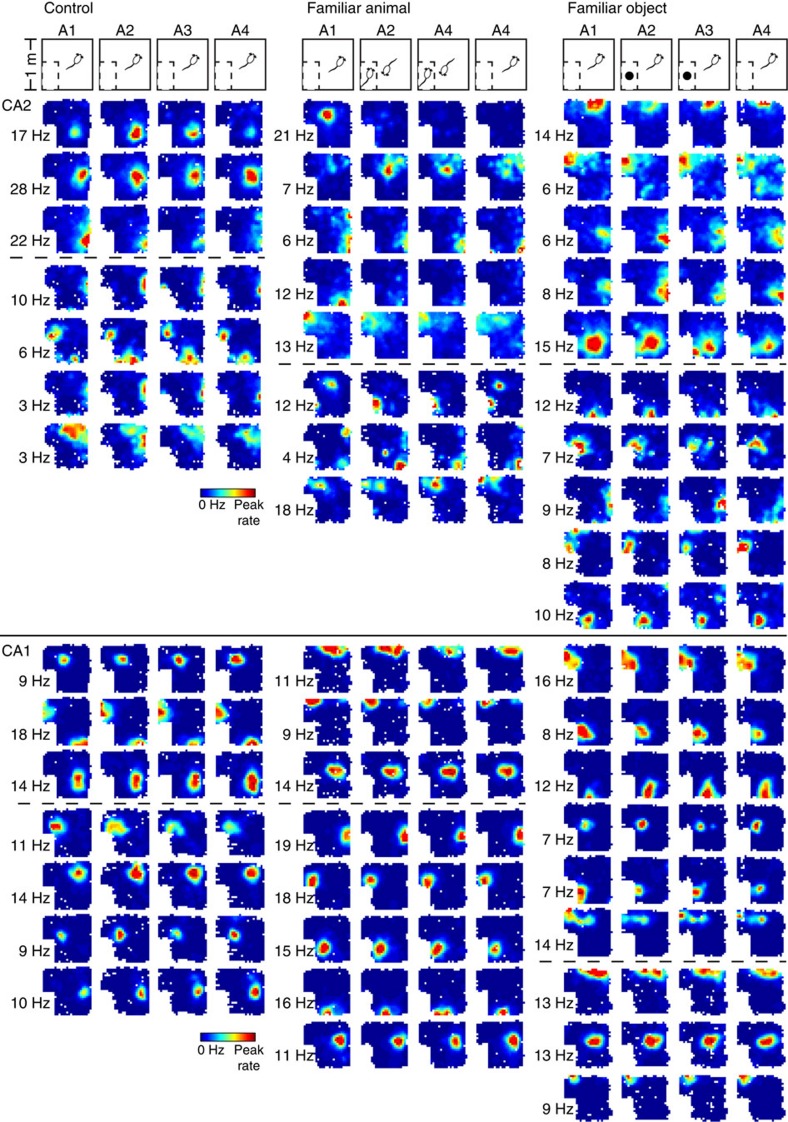
CA2 place cell remapping in response to social stimulation was replicated in a different strain of rats. Example colour-coded CA2 (upper) and CA1 (lower) place cell maps (dark blue indicates 0 Hz and dark red indicates peak rate for each cell) for the three behavioural paradigms illustrated above. Example maps are shown for all recorded CA2 and CA1 cells on a given day in two example rats for each condition (dotted horizontal lines indicate separation between cells from different rats). The peak rate for each cell is indicated to the left of its map. In the control condition (left column), rats explored an arena containing an empty cage and CA2 place cell maps changed gradually over time. In the social stimulation condition (middle column), a familiar rat was placed in the cage during the second and third behavioural sessions. Most CA2 place cells abruptly remapped in response to this social stimulation. The original maps did not return when the familiar rat was removed from the cage in the fourth recording session. In contrast, CA1 place cells exhibited stable maps across all sessions. In the control familiar object condition, a toy rat was placed in the cage during the second and third behavioural sessions. Neither CA1 nor CA2 cells exhibited consistent firing changes in response to the toy.

**Figure 5:**
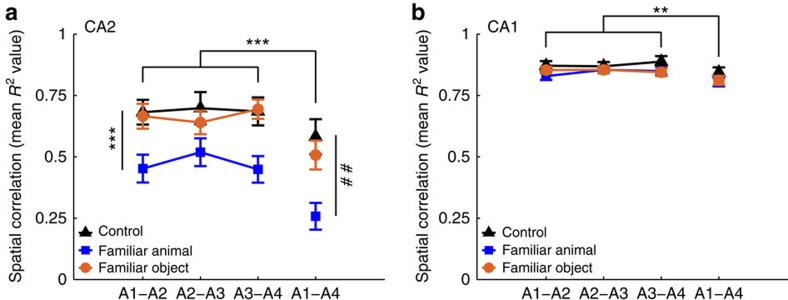
CA2 spatial correlations decrease with time and decrease further with social stimulation. Spatial correlations for CA2 (**a**) and CA1 (**b**) place cells for control, familiar animal and familiar object (stuffed animal toy) conditions. Spatial correlations were calculated between the first and second sessions (A1–A2), the second and third sessions (A2–A3), the third and fourth sessions (A3–A4) and the first and fourth sessions (A1–A4). For both CA2 and CA1, temporally adjacent sessions (A1–A2, A2–A3 and A3–A4) had significantly greater spatial correlations than temporally distant sessions (A1–A4). These significant effects of time (two-way ANOVAs: effect of time, CA2 (*n*=96 cells): F(1,378)=15.2, *P*<0.001; CA1 (*n*=253): F(1,1066)=11.9, *P*=0.001) are indicated with ***P<0.001 and **P=0.001. (**a**) CA2 place cells remapped in response to social stimulation, as evidenced by significantly lower spatial correlations in the social stimulation condition than in the other conditions (repeated-measures ANOVA: effect of condition, F(2,93)=8.5, ***P<0.001; control: *n*=11 neurons, familiar animal: *n*=47, familiar toy: *n*=38). In addition, for A1–A4 comparisons, spatial correlations were significantly lower for the social condition than the other conditions (one-way ANOVA: F(2,93)=7.4, ##*P*=0.001; *n*-values same as above). (**b**) CA1 place cells did not show significant remapping in response to social stimulation (repeated-measures ANOVA: effect of condition, F(2,250)=0.6, *P*=0.6, control: *n*=26 neurons, familiar animal: *n*=134, familiar toy: *n*=93). Error bars are s.e.m.

In a separate series of experiments in a different laboratory using a different strain of rat (Long–Evans) we obtained strikingly similar results (Figs 4 and 5). In these experiments, male rats with electrodes implanted in both CA2 and CA1 were placed into a familiarized open-field arena for four consecutive 20-min sessions. In the first (A1) and fourth (A4) sessions, no stimuli were presented. During the second (A2) and third (A3) sessions, animals were exposed to no stimulus (control), a familiar male rat (prior cage mate) or a familiar realistic-looking stuffed rat toy. The no stimulus control condition served to control for the passage of time^6^. The subject rat was familiarized to the familiar rat and stuffed toy in the testing conditions before recordings.

Consistent with previous reports^6^,^7^, spatial correlations were significantly lower in CA2 than in CA1, and spatial correlations in both CA2 and CA1 were significantly higher for temporally adjacent sessions (that is, A1–A2, A2–A3 and A3–A4) than temporally distant sessions (A1–A4) (two-way ANOVA for all CA1 and CA2 cells (*n*=349): effect of area, F(1,1392)=449.5, *P*<0.001; effect of time, F(1,1392)=38.1, *P*<0.001). A significant effect of time was also found for CA2 and CA1 cells tested separately (two-way ANOVAs: effect of time, CA2 (*n*=96 cells): F(1,37)=15.2, *P*<0.001; CA1 (*n*=253): F(1,1066)=11.9, *P*=0.001; Fig. 5). However, a stronger effect of time was observed for CA2 cells compared with CA1 cells across all behavioural conditions (two-way ANOVA: effect of area, F(1,343)=25.3, *P*<0.001; effect of condition, F(2,343)=1.9, *P*=0.2; interaction, F(2,343)=1.6, *P*=0.2; *n*-values same as above). Because of these effects, the correlations for temporally adjacent sessions were tested separately from A1–A4 correlations, and CA2 and CA1 cells were also assessed separately.

For CA2 neurons (control: *n*=11 neurons, familiar animal: *n*=47, familiar toy: *n*=38), spatial correlations for temporally adjacent sessions were significantly lower in the social stimulation (familiar animal) condition compared with the control and toy (familiar object) conditions (repeated-measures ANOVA: effect of condition, F(2,93)=8.5, *P*<0.001; Bonferroni *post hoc* tests: *P*=0.04 for control versus familiar animal and *P*=0.001 for familiar animal versus familiar object; Fig. 5a) but did not differ between control and toy conditions (Bonferroni *post hoc* test: *P*=1.0). A1–A2 spatial correlations were significantly different across conditions (one-way ANOVA: F(2,93)=6.1, *P*=0.003; *n*-values same as listed above), whereas A2–A3 correlations were not significantly different (one-way ANOVA: F(2,93)=2.7, *P*=0.07; *n*-values as above). These results suggest that CA2 place cells remapped in response to social stimulation, and that stable maps were formed in CA2 to represent social stimulation. Spatial correlations for A3–A4 were also significantly different across conditions (one-way ANOVA: F(2,93)=8.6, *P*<0.001; *n*-values as above). For A1–A4 spatial correlations, which compare two no-stimulus conditions that flank sessions containing either no stimulus or a familiar animal or object, spatial correlations were significantly lower in the social stimulation condition than in the other conditions (one-way ANOVA: F(2,93)=7.4, *P*=0.001; Bonferroni *post hoc* tests: *P*=0.05 for control versus familiar animal and *P*=0.002 for familiar animal versus familiar object; *n*-values as above). Taken together, these results suggest that CA2 place cells remapped in response to social stimulation and did not revert back to the original A1 maps during the A4 session. We did not detect a bias in the direction of place field shifts for any of the stimuli (*P*>0.05 for familiar animal, familiar toy and control conditions; Rayleigh test; Supplementary Fig. 12; *n*-values as above). Unlike CA2, CA1 place cells did not show significant remapping in response to social stimulation (repeated-measures ANOVA: effect of condition, F(2,250)=0.6, *P*=0.6, control: *n*=26 neurons, familiar animal: *n*=134, familiar toy: *n*=93; Fig. 5b).

## Discussion

In this study, we report several properties of hippocampal CA2 neurons and ensembles during exposure to different spatial and social contexts. We found that in agreement with recent studies^6^,^7^,^23^, CA2 neurons fire in place fields similar to neurons in CA1, but that they carry less spatial information than CA1 neurons. Consistent with CA2 neurons displaying place fields, neuronal activity in CA2 increased with spatial exploration, as measured by increases in average firing rate and IEG expression. Contrary to our hypothesis that was based on a recent report showing CA2 is essential for social recognition memory^11^, neuronal activity levels in CA2 were not further increased by social experience. In addition, cells targeted by CA2 efferents, the *Calb1−* neurons in CA1, were also not further modified by social experience as measured by IEG expression. However, we found that in CA2, representations of space remapped on presentation of novel and social changes to the environment. Furthermore, similar place cell experiments carried out in an independent laboratory yielded strikingly similar results, highlighting the robustness and reproducibility of these findings. Our findings indicate that CA2 place cells link novel and social information with representations of space.

These results generally support and further build on recent studies describing place field properties in CA2 (refs 6, 7). Using *in vivo* recordings in rats, both Mankin *et al*.^6^ and Lu *et al*.^7^ presented evidence that CA2 place fields are less stable than those in CA1 and CA3 when animals were put into familiarized contexts. Our data show that similarly, CA2 place cell maps change more than CA1 maps over successive 10-min epochs in the same context. Mankin *et al*.^6^ further assessed whether manipulating the shape of the context affected CA2 place fields and found that it did, but to a lesser extent than the passage of time over hours, suggesting that CA2 contextual coding is masked by the passage of time. Importantly, our experimental design controlled for the passage of time, so temporal encoding would have affected spatial correlations equally across exposures. Our results are consistent with recent findings that CA2 place maps change more across sessions than CA1 maps^6^,^7^, but we additionally found that CA2 neurons have the capacity for further updates in their representations, online, with the presentation of social or contextual changes to the environment. Notably, the changes in context that Mankin *et al*.^6^ and Lu *et al*.^7^ used were perhaps subtler than our change in context (border shape and box colour versus an intervening social or novel experience). In addition, Lu *et al*.^7^ assessed whether CA2 place field properties were modified across multiple novel contexts and found that the majority of CA2 neurons were active in all contexts, in contrast to CA1 and CA3, but that CA2 neurons globally remapped in novel contexts, similar to CA1 and CA3. These data are consistent with our findings in that CA2 place cells remap in response to novel contextual changes, and that a large proportion of remapping occurs by cells firing most in a different location.

Novelty and social experiences probably recruit inputs that are unique to CA2, such as those from the supramammillary nucleus^24^. CA2 neurons also express high levels of social neuropeptide receptors, including vasopressin 1b (ref. 16) and oxytocin^25^ receptors. Findings of a recent study using catFISH suggest that CA2 can detect novel changes to a learned context to a greater extent than CA1 or CA3 (ref. 9). Wintzer *et al*.^9^ found that when mice were exposed to two successive contexts, CA2 ensemble similarity decreased significantly more than CA1 and CA3 when the second context was identical to the first context but with two objects exchanged with two novel objects placed in the same configuration. Although catFISH is a very sensitive and powerful tool to dissect which cells are recruited into encoding a specific context, the technique is unable to distinguish specific changes to the encoding ensemble, such as rate remapping and shifts in place field firing locations^26^. Our studies, using recording of single neurons in awake behaving animals, complement the conclusions by Wintzer *et al*.^9^ that CA2 ensembles are indeed sensitive to updates to a context. We further determined how CA2 neurons respond to intervening novel or social experiences: by remapping spatial representations. Our findings therefore support the notion that CA2 plays a role in processing novelty and social information.

In addition, our finding that social exposure modifies CA2 place fields was independently replicated in a separate lab using a different strain of rat. Specifically, these experiments demonstrated that exposure to a familiar animal, but not a familiar stuffed toy animal, induced global remapping of CA2 firing but not CA1 firing. Surprisingly, when the rats were re-exposed to the empty arena following social exposure, the original maps did not return. One possible explanation is that CA2 stores yet another distinct representation of the environment to represent the memory that social interactions had previously occurred. Alternatively, a more complex interaction between effects of time and social stimulation on CA2 place maps may have taken place. Taking together the findings that novel but not familiar object exposure induced remapping, we suggest that CA2 may encode novelty similar to the way it encodes social interactions, which is supported by the catFISH findings described above^9^. Additional electrophysiological studies of novel versus familiar object exposure using identical procedures are needed to confirm whether remapping occurs specifically with novel but not familiar objects.

Our results also demonstrated that *Arc* expression in CA2 neurons increased in animals after they explored an open field arena but did not further increase with social exposure. Interestingly, whereas CA2 had a similar peak percentage of neurons with *Arc* foci as CA1 (∼25% in CA2 and 30% in CA1), *Arc* foci in CA2 were smaller under all conditions and time points tested. Single-molecule FISH is a very sensitive technique that affords substantially greater spatial resolution than traditional FISH methods. Thus, our observation that *Arc* foci were smaller in CA2 than in CA1 is indicative of lower rates of *Arc* transcription, consistent with the overall lower levels of *Arc* detected in CA2. This is probably because of CA2 neurons' robust ability to buffer and extrude calcium^27^, and/or high expression of STEP, an ERK1/2 phosphatase^28^, either of which would be predicted to prevent *Arc* transcription^29^,^30^,^31^.

Another interesting finding from our study was the higher percentage of neurons with *Arc* foci in CA2 at the 5-min time point than seen in CA1. We note that this may be due to CA2 having fewer and larger neurons that are more dispersed than those in CA1, so this percentage does not reflect the overall number of *Arc* foci+ neurons. However, one intriguing possibility is that transcriptional responses are indeed faster for a greater proportion of CA2 neurons than CA1 neurons. When taken together with the lower amount of *Arc* transcription in CA2, and that the percentage of *Arc* foci in CA1 surpasses the percentage of *Arc* foci in CA2 at the 15-min time point, a second scenario is that the higher percentage of *Arc* foci in CA2 at 5 min simply reflects earlier activation of more CA2 neurons. This may partly be explained by the stronger activation of CA2 by ECII than CA1 by ECIII^2^. A third possibility is that disynaptic inhibition of CA1 neurons by CA2 neurons may have resulted in a lower percentage of CA1 neurons with *Arc*+ foci at the 5-min time point^32^,^33^. Further studies are needed to parse out which mechanism(s) could account for this phenomenon.

We also observed a greater percentage of neurons that were positive for *Arc* foci in CA2 and CA1 in the control condition compared with the familiar and novel social conditions at 5 and 15 min. This is likely to have resulted from the social condition rats traversing the side of the arena with the social stimulus more frequently, at the expense of time spent in the rest of the arena, with the rats thus visiting fewer place fields. Notably, at the 15-min time point when rats had 10 min to explore the arena with the social stimulus (versus 3 min at the 5-min time point), the percentage of cells with *Arc* foci in the social conditions was more similar to that of the control condition.

Our study is the first to assess the function of CA2's direct target, CA1 deep neurons, during social processing. CA1 has only recently been appreciated as a bilayer of superficial, *Calb1*-expressing neurons and deep, *Calb1*-lacking neurons. Recent reports have shown using recordings from rat hippocampal slices *in vitro* that these populations are modulated via different and opposing inputs. Stimulation of CA3 evokes a predominantly excitatory response in superficial CA1 neurons and predominantly inhibitory response in deep CA1 neurons. Conversely, stimulation of CA2 evokes an inhibitory response in superficial CA1 and a biphasic excitatory response followed by an inhibitory response in deep CA1 (ref. 33). These data are consistent with an earlier report showing *in vitro* optogenetic activation of mouse CA2 afferents evokes an excitatory response predominately in the deep CA1 neurons^4^. As CA2 activity levels did not change with social exposure, we hypothesized that CA1 may have integrated a change in input weights (from CA2, CA3 and ECIII) that could have selectively shifted which population of CA1 neurons gets activated with social exposure. Contrary to our hypothesis, however, neither population of CA1 neurons was preferentially activated with social exposure as measured by *Arc* expression.

How might social and novel object exposure lead to remapping of place fields in CA2 neurons? Glutamatergic inputs onto CA2 neurons arise from neurons in layer II of medial and lateral entorhinal cortex, CA3 (ref. 34) and the dentate gyrus^4^. Input from grid cells, border cells and head direction cells in medial entorhinal cortex probably form the basis for place fields in hippocampal neurons^35^,^36^. In addition, CA2 place fields are likely to be influenced by input from CA3 neurons and dentate granule cells as well, both of which also display place fields^37^. Given that entorhinal cortical neurons realign but maintain their structure across environments^38^, whereas hippocampal neurons remap in different environments^39^,^40^, additional factors almost certainly influence place field remapping in hippocampal neurons^41^. Modulatory influences, such as changes in synaptic strength, may permit behaviourally relevant shifts in place field maps^42^. For example, CA2 neurons receive strong synaptic input from ECII, which is capable of long-term potentiation^2^. In contrast, CA2 is unique among the CA fields in that synaptic potentiation cannot be induced at intrahippocampal Schaffer collateral synapses under normal conditions^1^,^2^. However, specific neuromodulators, including vasopressin or oxytocin, contained in axons from the paraventricular nucleus of the hypothalamus that form synapses onto CA2 neurons^34^,^43^,^44^, permit synaptic potentiation of CA2 neurons^10^. An interesting possibility is that release of these peptides during exposure to social stimuli alters the balance of synaptic inputs onto CA2 neurons in a socially relevant manner to shift firing properties without increasing overall excitability, resulting in place field remapping. For remapping on novel object exposure, other neuromodulatory influences such as those from the supramammilary nucleus (SuM) input to CA2 neurons could also have an impact on the balance of synaptic weights and result in remapping^45^. SuM is known to be activated by novelty^46^ and to influence theta oscillations^47^, which are observed in hippocampal local field potentials (LFPs) during exploration of novel stimuli^48^. Conceivably, therefore, SuM inputs could play a role in modifying place field maps on novel object exposure. Given this unique complement of inputs, CA2 neurons are well positioned to signal the presence of a novel or social stimulus.

In summary, we have discovered a mechanism by which CA2 neurons could encode novel and social information: by modifying existing spatial representations. These data support existing evidence for how the hippocampus can integrate both declarative and spatial information, to create meaningful representations of experiences^49^,^50^.

## Methods

### Animals

Experiments were carried out in adult male Sprague–Dawley rats (250–350 g, 8–12 weeks; Charles River, Wilmington, MA). Rats were housed under a 12:12 light/dark cycle with access to food and water *ad libitum*. With the exception of data shown in Figs 4 and 5, animals were not food deprived during the experiments and experiments were conducted during the light cycle. All procedures were approved by the Animal Care and Use Committee of NIEHS and were in accordance with the National Institutes of Health guidelines for care and use of animals.

For the studies in Figs 4 and 5, experiments were carried out in four adult male Long Evans rats, weighing ∼450–550 g (6–9 months). Rats were maintained on a reverse light/dark cycle (lights off from 0800 to 2000, h) and tested during the dark phase. Rats recovered from surgery for at least 1 week before the start of behavioural testing. During the data collection period, rats were placed on a mild food deprivation regimen but did not drop below 90% of their free-feeding body weight. Rats had access to water *ad libitum*. The experiments were conducted according to the guidelines of the United States National Institutes of Health Guide for the Care and Use of Laboratory Animals under a protocol approved by the University of Texas at Austin Institutional Animal Care and Use Committee.

### Surgery

For *in vivo* electrophysiology experiments presented in Figs 1, 2, 3, nine rats underwent surgical implantation of multi-tetrode electrode bundles using sterile surgical techniques. CA1 pyramidal cells were targeted in five rats and CA2 pyramidal cells were targeted in five rats as well (dual recordings from both CA1 and CA2 were performed in one rat). Rats were anaesthetized with ketamine (100 mg kg^−1^) and xylazine (10 mg kg^−1^), and placed in a stereotaxic device. Four to six metal ground and anchor screws were secured in the cranium. A total of eight tetrodes bundled in medical grade tubing were implanted into either CA2 (−3.6 mm anteroposterior, 3.6 mm mediolateral and 3.5 mm dorsoventral from the bregma) or CA1 (3.6 mm anteroposterior, 2.0 mm mediolateral and 2.6 mm dorsoventral from the bregma), except for one animal in which two bundles of four tetrodes each were implanted into CA2 and the contralateral CA1. Tetrodes were constructed from 12.7 μm polyimide-coated nickel chromium (Rediohm-800) wire (Sandvic Materials Technology, Sandviken, Sweden), which were connected to a printed circuit board (San Francisco Circuits, San Francisco, CA) and miniature connector (Omnetics Connector Corporation, Minneapolis, MN). On the day of surgery, electrode tips were cut to the appropriate length and plated with gold to reduce electrode impedances to between 150 and 300 kΩ at 1 kHz by passing current though the wires, while the tips were immersed in gold solution (Neuralynx, Bozeman, MT, USA). Implanted electrodes were secured with dental acrylic. Following surgical implantation, animals were treated with carprofen (5 mg kg^−1^) to alleviate postsurgical pain.

For the experiments in Figs 4 and 5, rats were anaesthetized with isoflurane and surgically implanted, using sterile surgical techniques, with recording devices (‘hyperdrives'^51^) containing 14 independently movable tetrodes. Ten screws were secured in the skull and two of these screws were connected to the ground. The screws and the hyperdrive base were covered with dental cement to anchor the hyperdrive to the skull. Tetrodes were constructed from 17-μm polyimide-coated platinum iridium (90–10%) wire (California Fine Wire). In each rat, one of the tetrodes had all four wires connected to a single channel and this channel was used as a reference signal for differential recording and was placed in a quiet location at the level of the corpus callosum or higher. Another tetrode was lowered to the apical dendritic fields of CA1 and was used to monitor hippocampal electroencephalogram signatures as the rest of the tetrodes were slowly advanced to their target regions. The other 12 tetrodes were targeted towards cell body layers and were platinum plated to reduce single channel impedances to ∼150–300 kΩ at 1 kHz.

In the first rat, the stereotaxic coordinates (in mm) were −5.0 anteroposterior and 5.0 mediolateral; seven tetrodes hit CA1, one tetrode hit CA2 and three tetrodes hit CA3 (one tetrode did not reach its target). In the second rat, the coordinates were −5.0 anteroposterior and 4.4 mediolateral; six tetrodes hit CA1, one tetrode hit CA2 and five tetrodes hit CA3. In the third rat, the coordinates were −3.8 anteroposterior and 3.2 mediolateral; two tetrodes hit CA1, one tetrode hit CA2 and four tetrodes hit CA3 (five tetrodes did not reach their targets). In the fourth rat, the coordinates were −3.8 anteroposterior and 3.2 mediolateral; four tetrodes hit CA1, two tetrodes hit CA3 and one tetrode hit DG (five tetrodes did not reach their targets). The coordinates provided above did not exactly match with tetrode locations, because the diameter of the tetrode bundles was ∼1 mm. Tetrode placements were verified histologically at the end of each experiment. CA2 tetrodes were localized to the intermediate hippocampus in the first rat and the dorsal hippocampus in the second and third rats. CA1 tetrodes were localized to the dorsal hippocampus in all of the rats. Immediately following drive implantation, animals were treated with carprofen (5 mg kg^−1^) to alleviate postsurgical pain. Implanted rats were singly housed in cages (40 cm × 40 cm × 40 cm) constructed from clear acrylic and containing enrichment materials (for example, cardboard tubes and wooden blocks).

### Behavioural task

For experiments presented in Figs 1, 2, 3, beginning several days following surgery, neural signals were assessed every 1–3 days until single unit activity became apparent. While testing neural signals, animals were placed in the behavioural apparatus, which was a custom-built, open-topped arena made of opaque black plexiglass, ∼56 cm long, 54 cm wide and 77 cm high, with spatial cues on two opposing walls and an empty clear plexiglass cage insert (Supplementary Fig. 1). Animals were exposed to the arena for at least three 10-min sessions before the time that neural data were acquired. On the recording days, animals were placed in the arena, which was baited with fruit flavoured sucrose treats to encourage exploration for a period of 10 min. Next, either a familiar male rat or a novel male rat was placed in the insert (47 × 36 × 18 cm clear plexiglass cage insert with 0.5 inch holes every 2 cm, to allow for direct face, flank and ano-genital olfactory access) for a period of 10 min. On subsequent recording sessions, animals exposed to a novel rat were exposed to a familiar animal and vice versa. Novel animals had never been exposed to the subject animals before the time of testing and were housed in a separate colony room from the subject animals. Familiar animals were previous cage mates of the subject animals and were housed in the same colony room as subject animals after separation. The approximate time between singly housing and exposure of familiar animals to subject animals for testing was 1.5–2 weeks and familiar animals were encountered by subject animals in the arena context for the first time during recordings. During separate recording sessions, animals were also exposed to a novel object. Here, animals explored the open-field arena for 10 min with no stimulus and then a novel object was placed inside of the open-field arena. No border separated the novel object from the subject animal, but the objects presented a border by their shapes. Objects included Flexi-keys, Jump'n'Jacks and InnoDomes (all available from Bio-Serv). The order or presentation of novel or familiar rats or novel objects was randomized across animals.

For the experiments in Figs 4 and 5, tetrodes were gradually lowered to their target locations over ∼2–4 weeks. Behavioural testing began when place cell spikes emerged approximately at the proper depth for the region of interest, with amplitudes exceeding approximately five times the noise levels. Animals were tested in a 1 m × 1 m square open-topped arena, constructed from aluminium and wrapped with duct tape for shielding. During the control condition (‘Control'; see Fig. 4), a plexiglass rat housing cage filled with bedding material and covered with a filter top was placed in a corner of the arena. During the social stimulation condition (Familiar animal; see Fig. 4), an identical cage containing a familiar rat (male sibling and former cagemate) was placed in the same corner of the arena. During the toy rat control condition (Familiar object; see Fig. 4), an identical cage containing a realistic-looking stuffed toy rat was placed in the same corner of the arena. Behavioural sessions were 20 min in length and interspersed with 10-min rest sessions. During the rest sessions, the test rat was placed in a towel-lined flower pot on a pedestal. During behavioural testing, sweetened cereal or cookie crumbs were randomly scattered to encourage the rat to explore the arena.

Rats were familiarized to the empty testing enclosure for at least 3 days (three 20-min sessions per day) before behavioural experiments began. Rats were then familiarized to the social stimulation condition (that is, the condition in which the familiar rat was presented in the cage; see Familiar animal condition in middle panel of Fig. 4) for at least one day (four sessions per day, as shown in Fig. 4) before data collection, to ensure familiarity with the recording context.

### Neurophysiological data acquisition and behavioural tracking

For experiments presented in Figs 1, 2, 3, neural activity was transmitted via a 32-channel wireless 10 × gain headstage (Triangle BioSystems International, Durham, NC) and was acquired using the Cerebus acquisition system (Blackrock Microsystems, Salt Lake City, UT). Continuous LFP data were band-pass filtered at 0.3–500 Hz and stored at 1,000 Hz. Single unit data were sampled at 30 kHz and high-pass filtered at 250 Hz. All neurophysiological recordings were referenced to a ground wire connected to a ground screw. For behavioural tracking, the *X* and *Y* coordinates in space of a light-emitting diode present on the wireless headstage were sampled at 30 Hz using Neuromotive Software (Blackrock Microsystems) and position data were stored with the neural data. When comparing neuronal firing properties before and during exposure to social or novel object stimuli or control exposures with no stimuli presented, the two conditions occurred within a single recording session. Each data file that contained the two conditions were spike-sorted and files were separated into the two conditions only after the sorting was complete, thereby minimizing the possibility of misidentifying neurons due to multiple recording files. Following recordings, neurons were sorted into individual units by tetrode mode-based cluster analysis in feature space using Offline Sorter software (Plexon Inc., Dallas, TX). Autocorrelation and cross-correlation functions of spike times were used as separation tools. Only units with clear refractory periods and well-defined cluster boundaries were included in the analyses^52^. Pyramidal cells and interneurons were distinguished based on autocorrelation plots, waveforms and mean firing rates^53^,^54^. Putative interneurons based on these criteria were excluded from further analysis.

For the experiments in Figs 4 and 5, the hyperdrive was connected to a multichannel, unity gain headstage (HS-54, Neuralynx), the output of which was conducted via two lightweight tether cables through a multichannel slip-ring commutator to a data acquisition system that processed the signals through individual 24 bit AD converters (Digital Lynx, Neuralynx). Unit activity was band-pass filtered from 600 to 6,000 Hz. Spike waveforms above a threshold set by the experimenter (∼55–80 μV) were time-stamped and recorded at 32 kHz for 1 ms. LFP signals (one per recording tetrode) were band-pass filtered at 0.1–500 Hz and recorded continuously at a sampling rate of 2,000 Hz. Light-emitting diodes on the headstage were used to track the animal's movements at a 30-Hz sampling rate. The common reference signal for differential recording was duplicated using a breakout board (MDR-50 breakout board, Neuralynx) and recorded continuously against ground at a sampling rate of 2,000 Hz.

Following recordings, neurons were sorted into single units offline using graphical cluster-cutting software (MClust, A.D. Redish). Clustering was performed manually in two-dimensional projections of the multidimensional parameter space (consisting of waveform amplitudes, peak-to-valley ratios and energies). Autocorrelation and cross-correlation functions were used as additional separation tools. Each accepted single unit was required to exhibit well-defined cluster boundaries and a clear refractory period. Putative pyramidal cells were distinguished from putative interneurons on the basis of spike width, average firing rate and the presence of bursts. Putative interneurons, detected using these criteria, were excluded from further analyses.

### Electrode localization

For experiments presented in Figs 1, 2, 3, at the end of the physiological recordings, current was passed through the tetrodes that yielded pyramidal cell recordings (35 μA, 5 s). Two to four hours later, animals were deeply anaesthetized and perfused with 4% paraformaldehyde. Brains were postfixed for 24 h, submerged in 30% sucrose PBS and sectioned at 40 μm on a cryostat. CA1 tetrodes targeted the region of CA1 between the distal and proximal ends of CA1. If placement fell into the proximal or distal regions of CA1, the animal would not be included in the analyses. As electrode localization to CA2 can be difficult to delineate using Nissl stain alone^55^,^56^, some sections were immunolabelled for the CA2 marker STEP. In these cases, sections were incubated in anti-STEP antibody (Cell Signaling, 4817, 1:1,000 dilution, raised in mouse) followed by incubation in biotinylated anti-mouse secondary antibody (Vector), avidin–biotin complex and 3,3-diaminobenzidine, to identify peroxidase-stained CA2 in the area of the lesion.

For the experiments in Figs 4 and 5, at the end of the recording period, animals were deeply anaesthetized and perfused with 4% paraformaldehyde. For verification of tetrode locations, brains were cut coronally into 30 μm sections using a cryostat and the sections were subsequently stained with cresyl violet. All tetrode positions were identified by comparing locations across adjacent sections.

### Analyses

For experiments presented in Figs 1, 2, 3, a total of 252 CA2 neurons and 181 CA1 neurons were included from a total of nine rats that underwent both spatial and social experiments. For measurements of place field properties including number and size of place fields, spatial information and firing rate with behavioural state, 71 CA2 and 36 CA1 neurons were used. Of these neurons, 10 CA2 and 5 CA1 neurons were active (>0.2 Hz average firing rate) but did not reach criteria for displaying place fields; thus, 61 CA2 and 31 CA1 neurons were included in spatial analyses (that is, Fig. 1).

Of the total 252 CA2 neurons and 181 CA1 neurons, 181 CA2 and 145 CA1 neurons were used for analysis of changes in firing rate and spatial properties in response to social object of control exposure. Of the 181 CA2 neurons used for the social and object experiments, 31 were used for control exposure, 49 were used for familiar social exposure (37 for remapping), 55 were used for novel social exposure (54 for remapping) and 46 were used for object exposure. Of the 145 CA1 neurons, 28 were used for control exposure, 59 were used for familiar social exposure and 58 were used for novel social exposure (57 for remapping). These data were collected on days after the spatial experiments above. As we cannot say that we recorded from identical neurons on subsequent days, it is possible that a different population of neurons were used for spatial analyses and social analyses. Thus, the number of cells (*n*) is different for spatial verses social analyses. All graphs show mean and s.e.m. Data were analysed using Python 2.7 and Graphpad PRISM 6 software, and significance was determined using an *α*-level of 0.05.

For the experiments in Figs 4 and 5, 11, 47 and 38 CA2 neurons and 26, 134 and 93 CA1 neurons were included for Control, Familiar animal and Stuffed animal toy conditions, respectively. Data are shown as mean±s.e.m.

### Identification of place cells

For experiments presented in Figs 1, 2, 3, spatial firing rate distributions (‘place fields') for each CA2 and CA1 pyramidal cell were generated using Neuroexplorer version 4 software (Nex Technologies, Madison, AL). Briefly, the total number of spikes that occurred in each spatial location bin (2 cm × 2 cm bins) were summed and divided by the amount of time that the animal spent in that location. A Gaussian filter with s.d. of 2 cm was then applied to the data. If the animal spent <100 ms in a location, then that location appeared as a black square and was regarded as unvisited. The number of place fields for each neuron in the open-field arena was calculated by finding the peak firing rate pixel in the place field (>1 Hz), then drawing a perimeter of space in which firing rate was at least 80% of the peak firing rate. The process was then repeated until no further fields with peak rates above 1 Hz were found in the rate map. A total of 61 CA2 and 31 CA1 neurons displayed at least one place field in the open-field arena (10 CA2 and 5 CA1 neurons were active (>0.2 Hz average firing rate) but did not reach criteria for displaying place fields). Place field measurements were made from neurons before social or object exposure. Cells were categorized according to the number of place fields they displayed in the open-field arena and a histogram was generated to display the distribution of number of place fields per neuron in each area. The number of place fields for each neuron was also compared statistically across subregions using an unpaired *t*-test with Welch's correction (Supplementary Fig. 2).

For the experiments in Figs 4 and 5, spatial firing rate distributions (‘place fields') for each well-isolated place cell were constructed by summing the total number of spikes that occurred in each location bin (4 cm × 4 cm bins), divided by the amount of time that the animal spent in that location and smoothing using a Gaussian kernel with s.d. of 4 cm. Place fields across different sessions were compared using the spatial correlation measure described above. Pixels visited <150 ms in at least one of the sessions being compared were excluded from the analysis.

### Spatial information in bits/spike

For experiments presented in Figs 1, 2, 3, spatial information for each neuron was calculated using unsmoothed spatial maps with pixel bin sizes of 2 cm × 2 cm according to the following formula^57^,^58^:


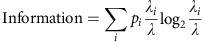


where *i* is an index over bins in the place field, *λ*_*i*_ is the mean firing rate in the *i*-th bin, *λ* is the mean firing rate for the entire field and *p*_*i*_ is the probability of the animal being in the *i*-th bin. Spatial information values were measured from the initial pre-social recording periods, which occurred after the animals had been exposed to the open-field arena for at least three 10-min sessions, but before any exposure of novel or familiar social stimuli. A Mann–Whitney test was used to compare spatial information content for CA1 (*n*=31 neurons) and CA2 neurons (*n*=61 neurons).

### Identification of behavioural states

Behavioural states were identified based on locomotor behaviour and the ratio of the power in theta band (5–11 Hz) to delta band (1–4 Hz) of LFPs, to eliminate any periods of sleep (slow wave sleep intervals characterized by the absence of movement according to video and tracking data, and low theta to delta power ratio^54^). Intervals when animals were traversing the environment and showed a high theta to delta ratio were classified as locomotor/exploratory periods. Intervals when the animal showed no traversal of the environment but tracking data revealed non-translational motion consistent with head movements were classified as quiet wakefulness. Locomotor and quiet awake intervals were isolated from recording data, and average and peak firing rates were determined using the rate histogram function in Neuroexplorer (v4). Sleep data collected in the open-field arena, distinct from periods of quiet wake and exploration, are not included in the current data set. Comparison of average and peak firing rates between CA1 and CA2 place cells during exploration was made with Mann–Whitney tests (*n*=61 CA2 pyramidal cells and 31 CA1 pyramidal cells; Fig. 1c,d). In addition, firing rates were compared for the same neurons during quiet wakefulness (Supplementary Fig. 3).

### Averaging firing rate during social exposure

To test the effect of social exposure on average firing rates, locomotor/exploratory periods were compared before and during social stimulation. Comparisons of average firing rates between CA1 and CA2, and between pre-social and social (either familiar or novel) conditions, were made using Wilcoxon matched-pairs signed-rank tests. Comparisons were made for all neurons regardless of whether they displayed a place field in the open-field arena or for which video analysis rather than tracking data were used to determine behavioural state before and during exposures (Fig. 2a,b; *n*=49 CA2 and 55 CA1 neurons for familiar social and *n*=55 CA2 and 58 CA1 neurons for novel social). In separate analyses, the same comparisons were made for only those cells that displayed place fields in the open-field arena (Supplementary Fig. 6; *n*=26 CA2 and 31 CA1 neurons for familiar social and *n*=19 CA2 and 31 CA1 neurons for novel social).

### Global remapping

Two methods the hippocampus uses to encode spatial information are global and rate remapping^59^,^60^. Remapping was assessed from recordings made before and during exposure to a social stimulus or a novel object placed in the arena. For global remapping, spatial correlations were calculated. Accordingly, Gaussian-smoothed spatial firing rate maps before and during each manipulation were compared by correlating the firing rate in each pixel between the two conditions and an *R*^2^ value for each neuron was calculated. Spatial correlations (Fig. 3b,d) were calculated for familiar social exposure (*n*=59 CA1 and 37 CA2 neurons), novel social exposure (*n*=57 CA1 neurons and 54 CA2 neurons) and novel object exposure (*n*=46 CA2 neurons). Example tracking and occupancy data are presented in Supplementary Fig. 11. Changes in peak firing rate were assessed by identifying the peak firing rate from unsmoothed place field maps during the pre-exposure period and during the exposure (social or object stimulation) period, and calculating the normalized change in firing rate according to the following formula: (peak rate in condition 2−peak rate in condition 1)/(peak rate in condition 2+peak rate in condition 1). Mean spatial correlations and normalized peak firing rate changes were compared with control values obtained by measuring the spatial correlation and change in peak firing rate between locomotor intervals with no social or object exposure during two adjacent 10-min sampling periods, to mimic the time between pre-social and social (or object) exposure (*n*=28 CA1 neurons and 31 CA2 neurons). Statistical significance was assessed using Kruskal–Wallis tests with Holm–Bonferroni corrections.

For the experiments presented in Figs 4 and 5, spatial correlation measures were not normally distributed. Thus, we transformed the data by log_10_(1−*x*), with *x* representing the spatial correlation value, before performing repeated-measures ANOVA, two-way ANOVA and one-way ANOVA, as indicated in the Fig. 5 legend. Transformed data did not differ from a normal distribution (Kolmogorov–Smirnov test, *P*=0.9). Bonferroni tests were used for *post hoc* comparisons.

To determine whether CA2 firing fields tended to shift in a particular direction (for example, towards a social stimulus), we calculated the shift of the centre of mass of the dominant place field (that is, the place field with maximal firing rate) for each place cell. We then assessed whether the directions of place field centre of mass shifts for each condition were uniformly distributed using the Rayleigh test (Supplementary Fig. 12).

### Spatial and social behavioural task for IEG expression

Rats were group housed for 1 week, to acclimate to their new environment, and then singly housed for the duration of the study. Rats were extensively handled on multiple days for 2 weeks before the start of the experiment, to control for stress during the task and to familiarize the rats to the experimenter. Rats were placed in the testing room for 1 h each day before the task began, to habituate to the testing room. After the 1-h habituation, individual rats were exposed to the arena described above for 10 min each day for 3 consecutive days, to habituate to the context. During habituation, the centre and four corners of the arena were baited with fruit-flavoured sucrose treats, to encourage exploration of the entire space. The arena was cleaned with 70% ethanol after each session. On the fourth day (test day), rats were randomized into three conditions using a random number generator (*n*=5 rats per condition): context alone, context plus former male cage mate (familiar), context plus novel oestrus female (novel). A novel oestrus female was selected to maximize the subject rats' interest. Power analyses were done *a priori*, to determine animal numbers per condition (80–90% statistical power using ANOVA followed by one-sided *t*-tests at the 0.05 level of significance comparing pairs of conditions). After a rat was habituated to the room for at least 1 h, it was placed in the arena and a familiar or novel rat was placed in the clear insert described above. For the context-only condition, nothing was placed into the insert. The rats were allowed to explore the context for either 3 min (5 min time point) or 10 min (15 and 30 min time points) and then placed back into their home cage until they were killed by rapid decapitation without anaesthesia at a total time of 5, 15 or 30 min from the onset of exploration. Each time point was performed with a separate cohort of animals and the 30-min time point was performed twice, because one brain was not usable for *in situ* during the first cohort (*n*=9). HCC animals were taken directly from their cage at different hours throughout the testing day and immediately killed (*n*=10). Brains were removed and flash frozen in isopentane cooled to −20 °C in a dry ice/ethanol bath.

### Oestrous cycle tracking

Adult female Sprague–Dawley rats were group housed in a different room from the subject rats used above, to limit exposure to scents and vocalizations. A basic voltmeter used to read membrane resistance was modified to non-invasively determine oestrous phase in rats^61^. This method was verified in-house to read a higher membrane resistance (300–400 kΩ) on the day(s) when female rats were in oestrus as compared with lower readings (100–200 kΩ) during the three other phases (dioestrus, proestrus and metoestrus). Readings were taken twice daily for multiple days before test day. In all cases, vaginal smears and cytology were used to confirm a positive oestrus reading from the voltmeter.

### Multiplexed single-molecule FISH

Brains were embedded in optimal cutting temperature compound and sectioned on a cryostat at 20 μm and processed for single-molecule FISH according to the RNAscope Fluorescent Multiplex kit instructions (Advanced Cell Diagnostics, Hayward, CA). RNAscope technology allows for the detection of single RNA molecules by way of its zz oligo pair design and DNA-based amplification methods^62^. The following probes were used with the RNAscope fluorescent multiplex reagent kit: Rn-Arc-3p (catalogue number 317076), Rn-Egr1-C2 (318576-C2), Rn-Pcp4-C3 (318586-C3) and Rn-Calb1-C2 (417551-C2). The *Arc* probe was designed against nucleotides 1,519–2,621 in the mRNA sequence, the *Egr1* probe was designed against nucleotides 162–1,333, the *Pcp4* probe was designed against nucleotides 3–518 and the *Calb1* probe was designed against nucleotides 144–1,270.

### Image acquisition and quantification

All images were acquired on a Zeiss 710 meta confocal microscope using a × 40 oil-immersion lens. The image acquisition and quantification were done under blind conditions. Our acquisition parameters were set using 3plexed negative controls (complementary DNA probes against bacterial RNAs not present in mouse tissue) in each of the three channels (fluorescein isothiocyanate, CY3 and CY5) so that any signal above the level of background was acquired. Area CA2 borders were identified using *Pcp4* labelling and area CA1 was identified using its defined anatomical location. Each image was analysed in its unprocessed form and no thresholding was done before quantification. All statistical analyses were carried out using Graphpad PRISM 6 software and significance was determined using an *α*-level of 0.05.

### Time-course experiment

One 212 μm × 212 μm region per subfield per hippocampus (dorsal) was imaged in *z*-stack using 1-μm steps. Images were processed using FIJI software (NIH v.1.47). *Arc* and *Egr1* foci were counted in cells as previously described^21^ and 8,791 cells were included in the analyses. Briefly, foci were counted in cells that were diffusely labelled with 4,6-diamidino-2-phenylindole (DAPI) and positioned within the cell body layer. Cells that showed unusually bright DAPI staining, characteristic of glia, were excluded from quantification. Cells were designated as positive for *Arc* or *Egr1* foci when they: (1) showed at least one characteristic bright spot of fluorescence within the nuclei and (2) the bright spot appeared in at least three individual *z*-slices. The data are presented as average per cent of total nuclei with *Arc* or *Egr1* foci±s.e.m. Owing to the difference in cell size and spacing across subregions, the CA2 region had ∼60 cells per image and CA1 had ∼90 cells per image. Data from the left and right hippocampi were not statistically different and were included in statistical analyses as one average per animal.

### Calbindin-1 experiment

Three 212 μm × 212 μm dorsal CA1 regions (proximal, centre and distal) were imaged in *z*-stack per hippocampus for the 15-min time point cohort only. Nuclei were designated as positive for *Arc* foci (as defined above) and/or *Calb1* positive or negative. In addition, the total number of *Calb1*-positive and -negative cells were counted and the data are presented as average per cent *Calb1*+ or − cells expressing *Arc* foci. A total of 7,941 cells were included in the analyses. As proximal, centre and distal CA1 regions were included in these analyses, we report the percentage of cells with *Arc* foci from these three CA1 regions in Supplementary Fig. 8. Differences in the percentage of cells with *Arc* foci in proximal, centre and distal regions of CA1 were statistically different; however, as no significant differences were detected across conditions, the three CA1 regions were collapsed within each animal and graphs are presented as an average per cent across rats ±s.e.m. per condition (*n*=3–5 rats per group, *n*=5 Context and HCC, and *n*=6 when Familiar and Novel are combined as Social condition). We also measured the average nuclear *Arc* fluorescence per *Arc* foci+cell using Zen Blue 2012 software (Carl Zeiss). The *Arc* foci+nuclei were identified and outlined using the DAPI channel with the contour tool and saved as a region of interest. The nuclear region of interest was used to measure the fluorescence intensity in the *Arc* channel for each *Arc* foci+nuclei and then classified as *Calb1* positive or negative. The data are presented as average *Arc* nuclear fluorescence per cell for *Arc+* cells only.

### Double label immunofluorescence

Forty-micrometre-thick vibratome-cut brain sections from perfused rats were rinsed in PBS and blocked for at least 1 h in 3% normal goat serum/0.01% Tween 20. Sections were incubated in the following primary antibodies: mouse anti-regulator of G-protein signalling 14 (UC Davis/NIH NeuroMab Facility, AB_10698026, 1:1,000) or mouse anti-STEP (Cell Signaling, 4817, 1:500) and rabbit anti-PCP4 (SCBT, sc-74186, 1:500). Antibodies were diluted in blocking solution and sections were incubated for 24 h. After several rinses in PBS/Tween, sections were incubated in secondary antibodies (Alexa goat anti-mouse 488 and Alexa goat anti-rabbit 568, Invitrogen, 1:500) for 2 h. Finally, sections were washed in PBS/Tween and mounted under ProLong Gold Antifade fluorescence media with DAPI (Invitrogen).

## Additional information

**How to cite this article:** Alexander, G. M. *et al*. Social and novel contexts modify hippocampal CA2 representations of space. *Nat. Commun.* 7:10300 doi: 10.1038/ncomms10300 (2016).

## Supplementary Material

Supplementary Information Supplementary Figures 1-12, Supplementary Table 1, Supplementary Methods and Supplementary References
