# Supplementary material for: Social and novel contexts modify hippocampal CA2 representations of space
Source: Nat Commun. 2016 Jan 25;7:10300. doi: 10.1038/ncomms10300 (PMC4737730; doi:10.1038/ncomms10300)
Supplement: Supplementary Information — Supplementary Figures 1-12, Supplementary Table 1, Supplementary Methods and Supplementary References [file ncomms10300-s1.pdf]

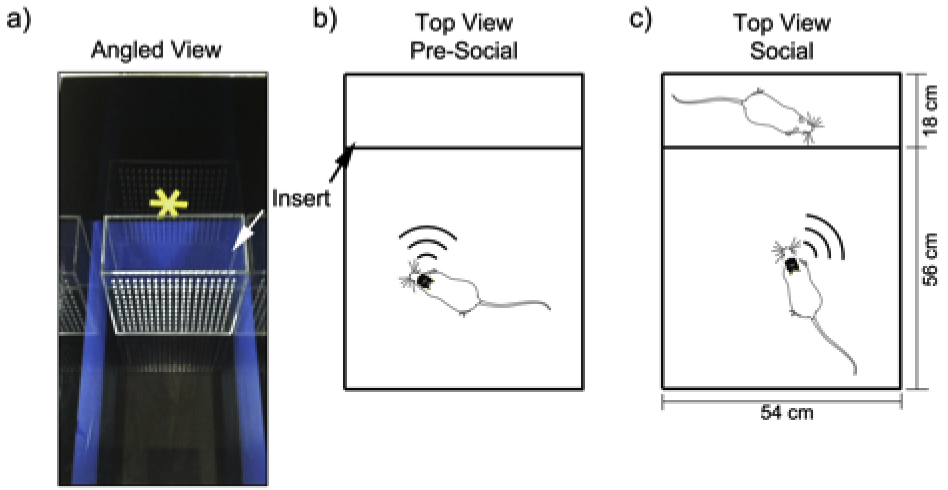

Supplementary Figure 1. Photo and schematic diagrams of open field arena used for these studies (except Figures 4-5 and Supplementary Figure 12d-f). (a) Photo of the inside of the area taken at an angle to demonstrate the insert where the novel or familiar rat would be placed during social experiments. The plexiglass cage insert had ½-inch holes every 2 cm to allow for direct face, flank and ano-genital olfactory access. (b-c) Schematic diagrams of open field arena shown from a top view demonstrating the area that the subject rat was free to explore. During non-social experiments, no rat was placed in the insert. During social experiments, a novel or familiar rat was placed in the insert. The subject rat in (b) and (c) is shown with a wireless transmitting headstage and semicircles represent wirelessly transmitted neural signals. For IEG experiments, rats were not implanted with electrodes and did not have wireless headstages.

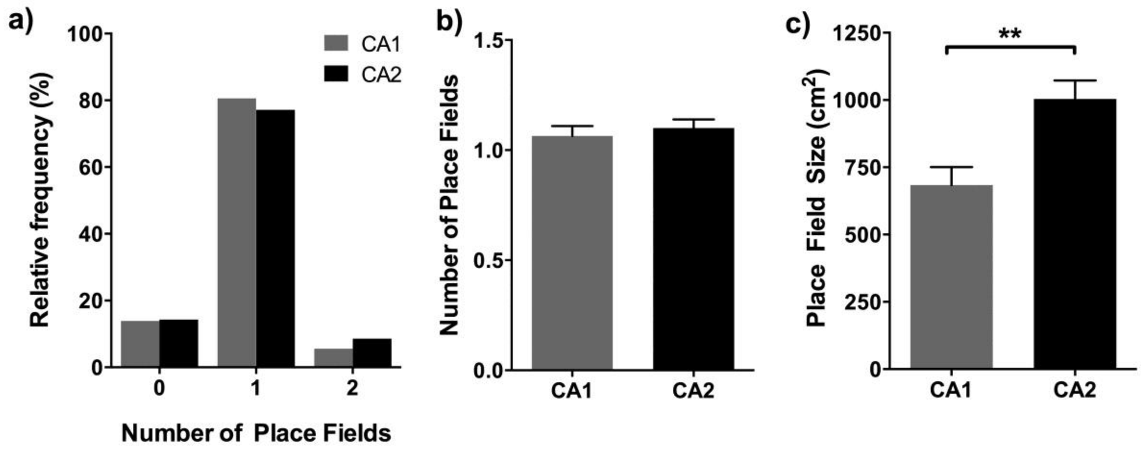

Supplementary Figure 2. Histogram of numbers of place fields per neuron **(a)**, mean numbers of place fields per neuron, including neurons with no place fields **(b)**, and mean place field sizes for neurons with place fields **(c)** for CA1 (grey bars) and CA2 (black bars). There was no significant difference in the number of place fields per neuron between CA2 and CA1 (median(CA1)=1, median(CA2)=1, U=913.5, $p>0.05$, one-tailed unpaired Mann-Whitney test, n=61 CA2 neurons and 31 CA1 neurons), a result that differs from previous reports[1,2] likely due to the smaller size of the open field arena in the current study. CA2 neurons had significantly larger place fields than CA1 neurons (median(CA1)=577.7, median(CA2)=928.0, U=678, $p<0.01$, one-tailed unpaired Mann-Whitney test, n=61 CA2 neurons and 31 CA1 neurons) in the same open field arena. **$p<0.01$. Error bars are SEM.

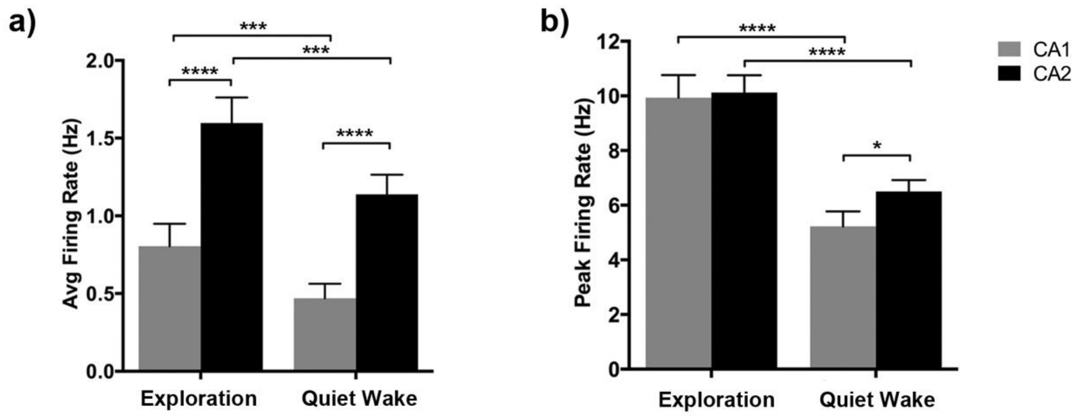

Supplementary Figure 3. Average **(a)** and peak **(b)** firing rates for CA1 and CA2 place cells during exploration and quiet wakefulness. **(a)** CA2 place cells had significantly higher average firing rates than CA1 place cells during both exploration (CA1 vs CA2 median (median, CA1)=0.5146, median(CA2)=1.354, U=468, p<0.0001, Mann-Whitney test, n=61 CA2 neurons and 31 CA1 neurons) and quiet wakefulness (i.e., brief periods of immobility that occurred between exploratory epochs; median(CA1)=0.2400, median(CA2)=0.9086, U=413, p<0.0001, Mann-Whitney test, same n values as above). Both CA1 place cells and CA2 place cells had a higher average firing rate during exploration than during quiet wakefulness (CA1: median(exploration)=0.5146, median(quiet wake)=0.2400, W=338.0, p<0.001; CA2: median(exploration)=1.354, median(quiet wake)=0.9086, W=935.0, p<0.001, Wilcoxon sign rank tests, same n values as above). **(b)** CA2 place cells had significantly higher peak firing rates (spikes/sec) than CA1 place cells during quiet wakefulness (median(CA1)=4, median(CA2)=6, U=681.0, p<0.05, Mann-Whitney test, same n values as above) but did not have significantly higher peak firing rates during exploration (median(CA1)=9, median(CA2)=9, U=860, p>0.05, Mann-Whitney test, same n values as above). Both CA1 and CA2 place cells had significantly higher peak firing rates during exploration than during quiet wakefulness (CA1: median(exploration)=9, median(quiet wake)=4, W= 421.0, p<0.0001; CA2: median(exploration)=9, median(quiet wake)=6, W=-1220.0, p<0.0001, Wilcoxon sign rank tests, same n values as above). * p<0.05, *** p<0.001, **** p<0.0001. Error bars are SEM.

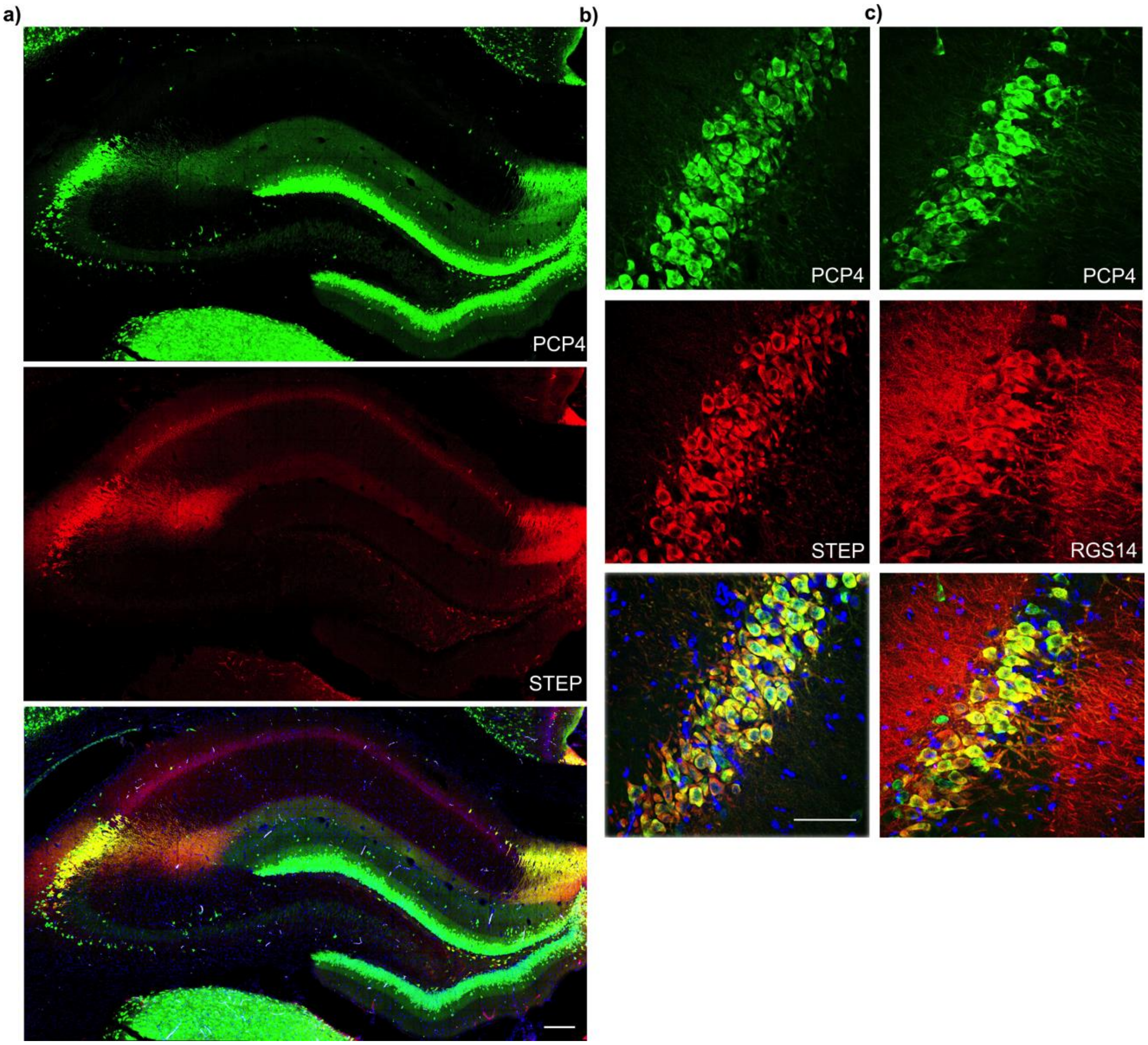

Supplementary Figure 4. Double label immunofluorescent staining with CA2 markers. **(a)** Low magnification images of PCP4 and STEP immunofluorescence colocalizing in dorsal rat hippocampus. **(b-c)** High magnification images of CA2 fluorescence colocalization when detecting PCP4 and STEP **(b)** or RGS14 **(c)**. Scale bars = 200 μm (a), 50 μm (b).

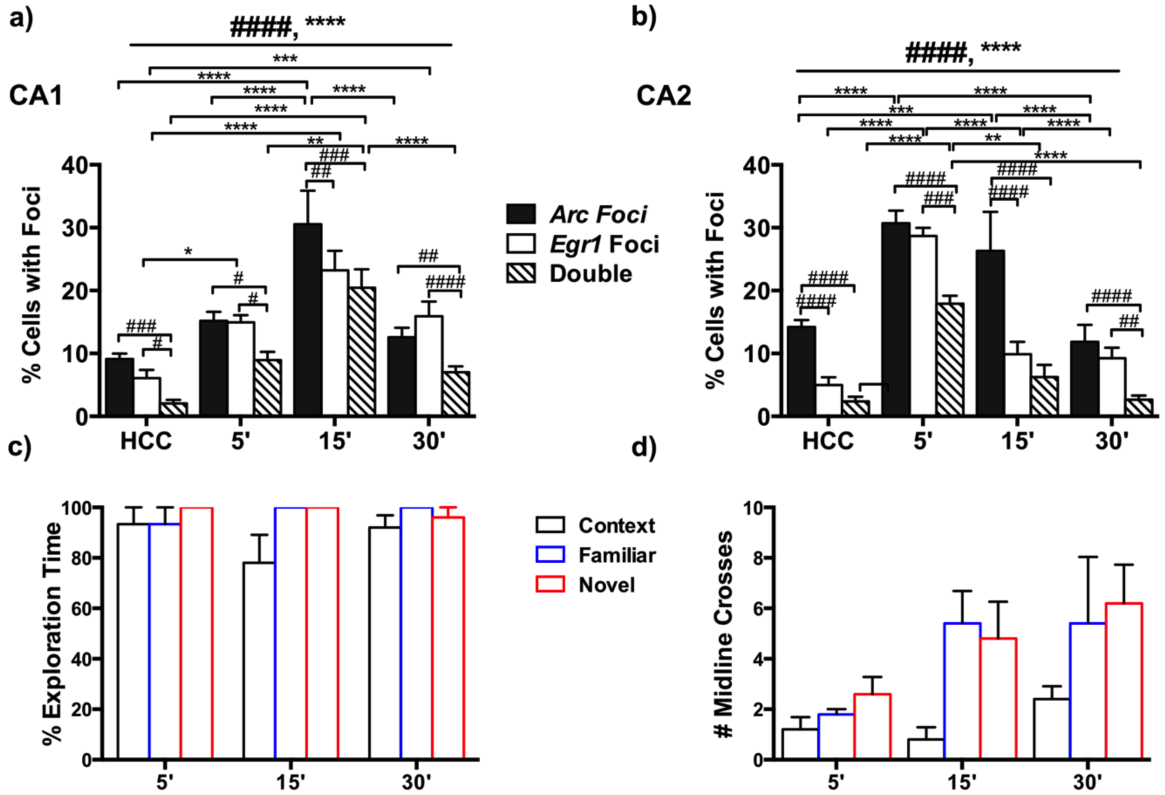

Supplementary Figure 5. Percentage of cells expressing *Arc* foci, *Egr1* foci or both across time for CA1 (**a**) and CA2 (**b**). There was an overall effect of time and group for both subregions (**CA1:** main effect of time F (3, 25) = 24.26 p<0.0001; main effect of group F (2, 50) = 28.04, p<0.0001, two-way paired ANOVA with Bonferroni post hoc tests; **CA2:** main effect of time F (3, 25) = 26.95, p<0.0001; main effect of group F (2, 50) = 70.20, p<0.0001, two-way paired ANOVA with Bonferroni post hoc tests). Note that we were unable to robustly detect *Egr1* foci in CA2 at the later time points using RNAscope. (**c**) Behavioral data comparing percent exploration time and (**d**) number of midline crosses for the 5 and 15 and 30-minute time point cohorts. Note that the total exploration time was 3 min. for the 5 min. time point and 10 min. for the 15 and 30 min. time points. Also note the increase in midline crosses for the social conditions. Based on experimenter notes during the behavior, rats with social exposure tended to have quicker exploration speeds and spent an increased amount of time exploring the area of the arena where the social stimulus was present. #,*p<0.05; ##,**p<0.01; ###,***p<0.001; ####,****p<0.0001. N=10 rats for HCC, 5 rats for 5' and 15' time points and 9 rats for 30' time point. Error bars are SEM.

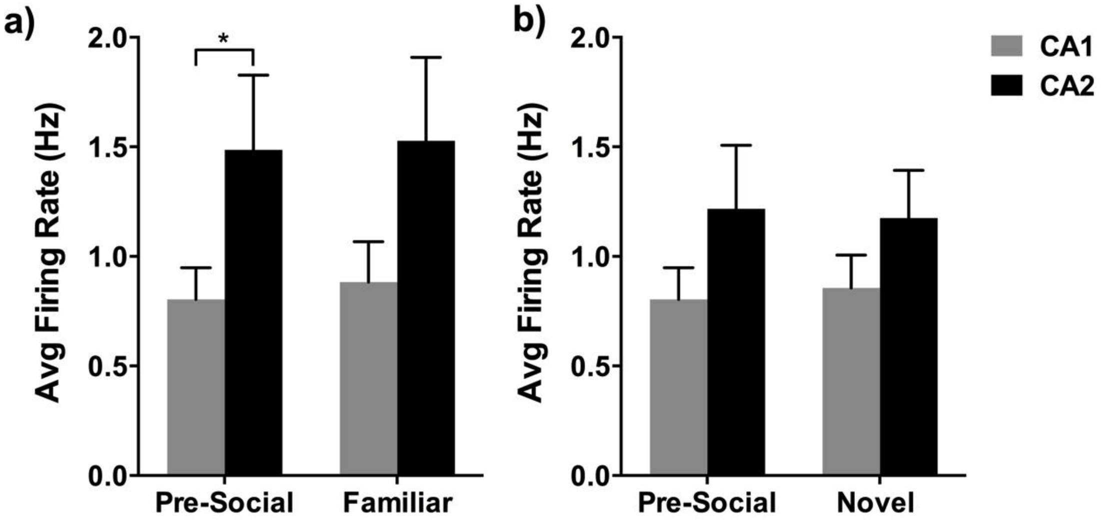

Supplementary Figure 6. Average firing rates of CA1 and CA2 neurons before and during exposure to a (**a**) familiar or (**b**) novel rat. Only neurons displaying place fields in the open field arena were included in the analysis. No differences in average firing rates were detected for CA2 neurons during familiar rat exposure (median(pre-social)=0.812, median(familiar)=0.878, W=-5.000, p>0.05, n=26 CA2 place cells) or novel rat exposure (median(pre-social)=0.794, median(familiar)=0.787, W=10.00, p>0.05, n=19 CA2 place cells, Wilcoxon matched-pairs signed rank test) or for CA1 neurons during familiar rat exposure (median(pre-social)=0.515, median(familiar)=0.540, W=106, p>0.05, n=31 CA1 place cells) or novel rat exposure (median(pre-social)=0.5146, median(familiar)=0.4864, W=150.0, p>0.05, n=31 CA1 place cells Wilcoxon matched-pairs signed rank test). Comparisons of CA1 vs CA2 firing rates for each of the four conditions shown revealed a significant increase in average firing rate for CA2 over CA1 during the pre-familiar social condition only (median(CA1 pre-social)=0.5146, median(CA2 pre-social)=0.8120, U=269, p<0.05, n=31 CA1 place cells and 26 CA2 place cells). All other comparisons did not reach significance. *p<0.05. Error bars are SEM.

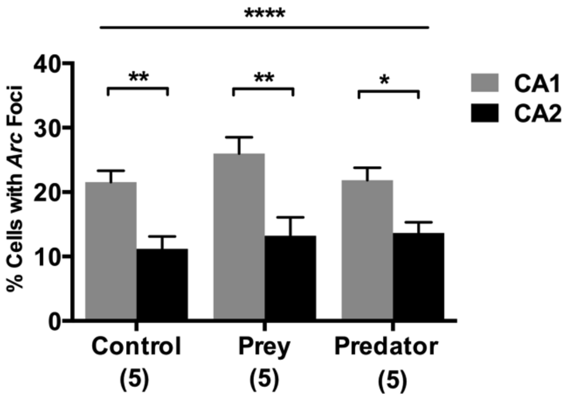

Supplementary Figure 7. Social behavior in rodents is heavily dependent on olfaction, and behavioral responses to social aggression are impaired in *Avpr1b* knockout mice[3]. We therefore investigated also whether other survival-relevant olfactory cues (predator or prey scents) could activate CA2 neurons independent of exploration. Predator (fisher cat urine) or prey (soiled mouse bedding) scents were presented in an egg carton inside the rats' home cages and animals were sacrificed 15 minutes following initial exposure, at the time of typical peak *Arc* expression. The figure shows the percentage of cells expressing *Arc* foci across behavioral conditions. There was an overall effect of subregion and no effect across groups ($F(1,12) = 45.32$, $p<0.0001$, two-way, paired ANOVA with Bonferroni post hoc tests, N=5 rats per condition). These data demonstrate that survival-relevant stimuli do not increase CA2 neuronal activity as assessed by *Arc* expression. *$p<0.05$; **$p<0.01$. Error bars are SEM.

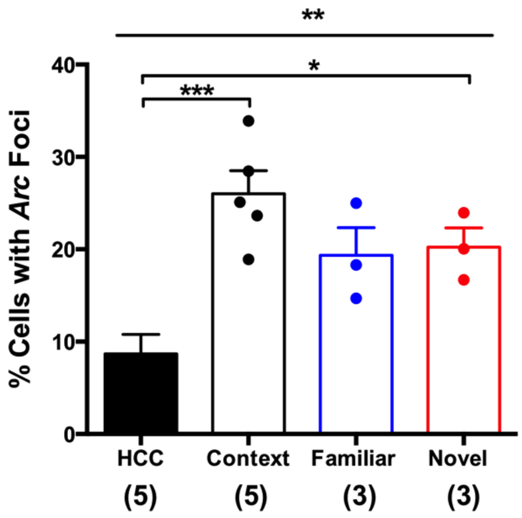

Supplementary Figure 8. Percentage of CA1 cells expressing *Arc* foci across behavioral groups at the 15' time point (maximal nuclear *Arc* expression) for the *Calb1* study. There was an overall effect of behavioral group ($F(3,12)=10.44$, $p= 0.0012$, one-way ANOVA with Bonferroni post hoc tests) HCC vs. Familiar approached significance (adjusted p value $p=0.07$). For x-axis labels, numbers in parentheses indicate number of rats per group. *$p<0.05$; **$p<0.001$; ***$p<0.001$. Error bars are SEM.

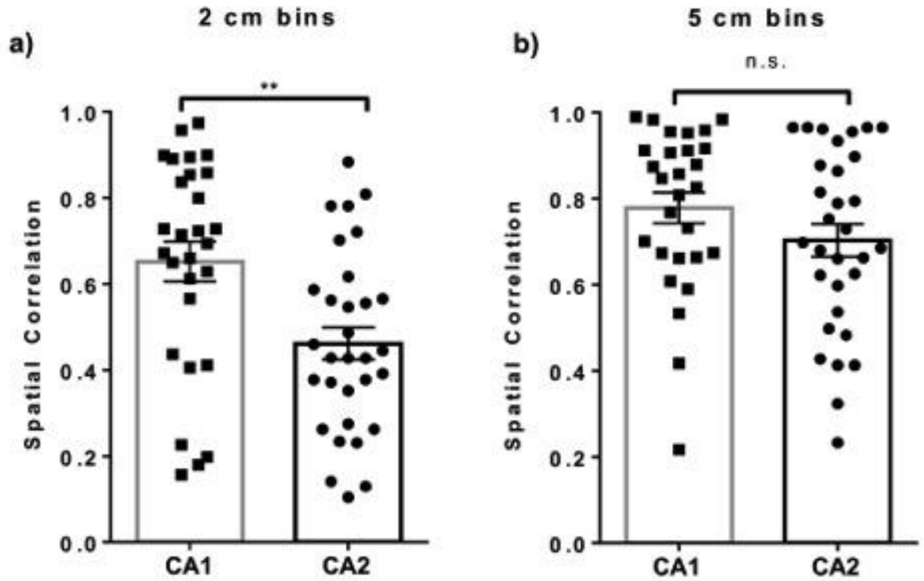

Supplementary Figure 9. Control spatial correlation values for CA1 and CA2 neurons over successive 10-minute epochs using a spatial bin size of either 2 cm (a) or 5 cm (b), which was the bin size used by Mankin *et al*. (2015). Bars represent the population means (+/- SEM), and individual data points comprising the means are shown. With a 2-cm bin size (a), control spatial correlation values for CA1 were significantly greater than those for CA2 (median(CA1)=0.704, median(CA2)=0.429, U=230, $p<0.01$(**), Mann-Whitney test, n=28 CA1 and 31 CA2 neurons). However, when using a bin size of 5 cm (b), we did not observe a significant difference in control spatial correlation values between CA1 and CA2 (median(CA1)=0.831, median(CA2)=0.698, U=345, $p>0.05$ (n.s.), Mann-Whitney test, same n values as above).

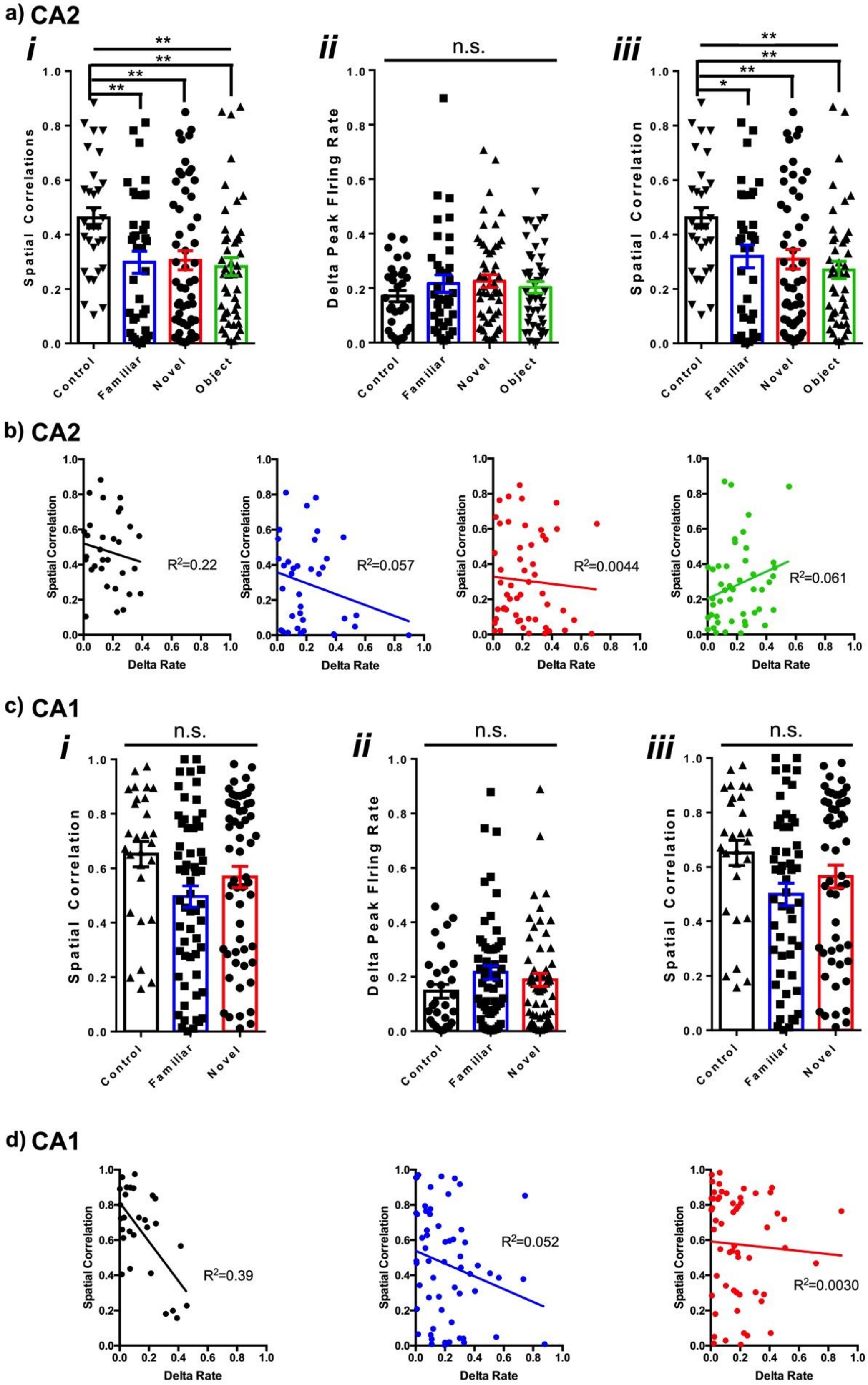
a) CA2
i
ii
iii
Spatial Correlations
Delta Peak Firing Rate
Spatial Correlation
n.s.
Control
Familiar
Novel
Object
b) CA2
Spatial Correlation
Delta Rate
R2=0.22
R2=0.057
R2=0.0044
R2=0.061
c) CA1
Control
Familiar
Novel
d) CA1
R2=0.39
R2=0.052
R2=0.0030

Supplementary Figure 10. Spatial correlations and peak firing rate changes between successive behavioral sessions (see Fig. 3a) for individual CA2 (**a-b**) and CA1 (**c-d**) neurons during either control (i.e., when no stimulus was presented to the animal), or exposure to a familiar or novel social stimulus or a novel object. Symbols represent individual neurons for all plots and bars represent population means (+/- SEM) in (a) and (c). (**a**) ***i***, Spatial correlation values for the entire population of CA2 neurons. Spatial correlation values were significantly decreased during familiar and novel social exposure as well as novel object exposure. (** $p<0.01$, Kruskal-Wallis test with Holm-Bonferroni correction, control: n=31 neurons, familiar social: n=37, novel social: n=54). ***ii***, Delta peak firing rate values for all neurons. There was no effect of exposure on delta firing rates in CA2 (n.s.= not significant, Kruskal-Wallis test, $p>0.05$, same n values as above). ***iii,*** Spatial correlations for only those CA2 neurons with unaffected firing rates, as defined by delta firing rates less than 0.5 (selected based on control data). Spatial correlation values remained significantly decreased during familiar and novel social exposure as well as novel object exposure ($p<0.01$, Kruskal-Wallis test with Holm-Bonferroni correction, control: n=31 neurons, familiar animal: n=34 neurons, novel animal: n=51 neurons, novel object: n=45 neurons). (**b**) Correlations between delta peak firing rate and spatial correlation values for all CA2 neurons during control (black), familiar social (blue), novel social (red) and novel object (green) experiments. Correlation coefficients for each plot are shown on the plots. (**c**) ***i***, Spatial correlation values for all CA1 neurons upon social exposure were not significantly different from control exposure (no stimulus) ($p>0.05$; Kruskal-Wallis test, n=28 neurons, familiar animal: n=59 neurons, novel animal: n=57 neurons). ***ii***, Delta peak firing rate values for all CA1 neurons were also not significantly different from control values ($p>0.05$; Kruskal-Wallis test, same n values as above). ***iii,*** Spatial correlations for only those CA1 neurons with unaffected firing rates, as defined by delta firing rates less than 0.5 (selected based on control data). Spatial correlation values during social exposure were not significantly different from control values ($p>0.05$; Kruskal-Wallis test, n=28 neurons, familiar animal: n=53 neurons, novel animal: n=53 neurons). (**d**) Correlations between delta peak firing rate and spatial correlation values for all CA1 neurons during control (black), familiar social (blue), and novel social (red) experiments. Correlation coefficients for each plot are shown on the plots.

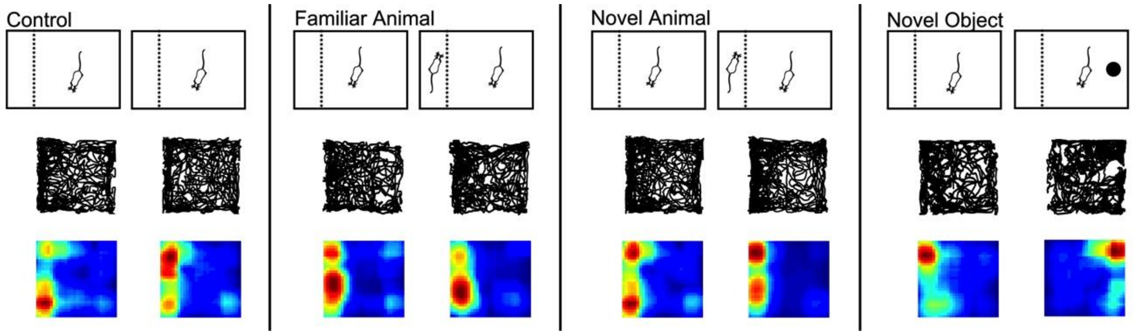

Supplementary Figure 11. Example tracking and occupancy data for each of the four conditions tested for place field remapping. The top row shows schematic diagrams of each of the two sessions compared for each condition, the middle row shows tracking data for the subject rat during each session, and the bottom row shows occupancy data for each of the sessions. Red colors indicate more time and blue colors indicate less time.

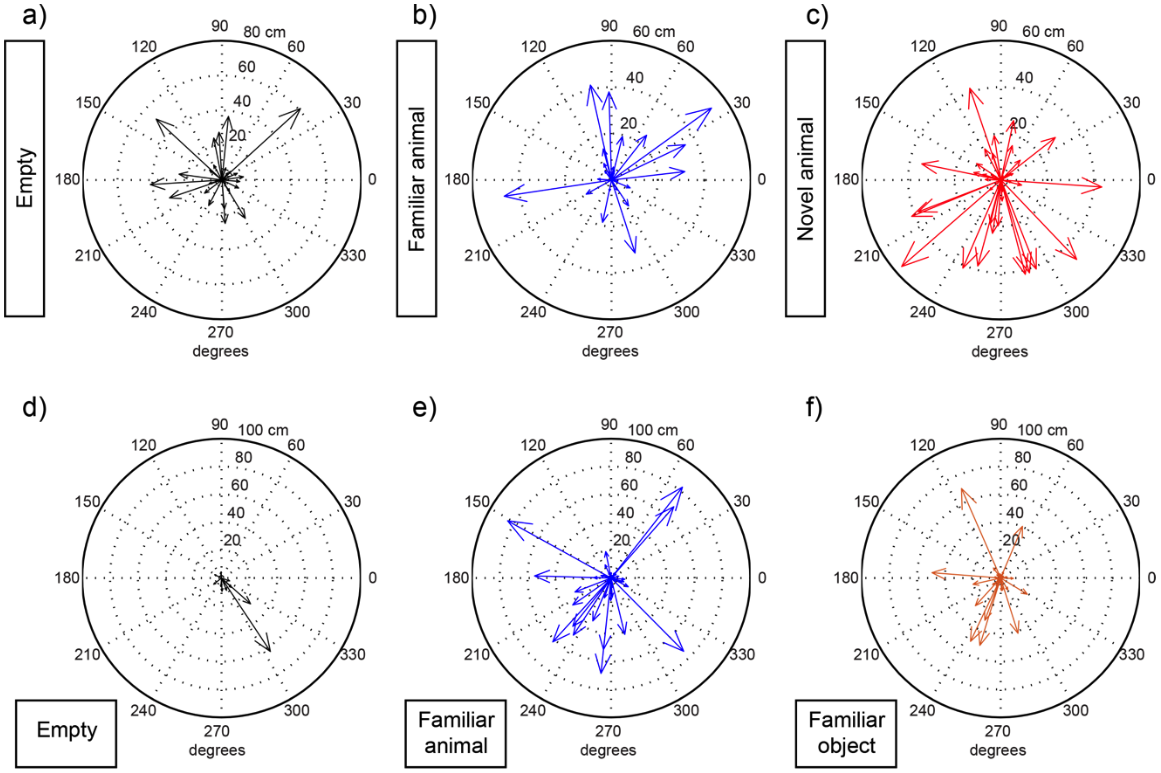

Supplementary Figure 12. Polar plots resulting from center of mass analyses of data presented in Figure 3 (**a-c**) or data presented in Figure 4 and 5 (**d-f**) to address whether there was a bias in the direction of place field shifts. Center of mass analyses were applied to place field data for before and during presentation of no stimulus (**a, d**), a familiar rat (**b, e**), a novel rat (**c**), or a familiar object (**f**). No significant shift in place fields was found with this method of analysis under any conditions tested ($p>0.05$ for all conditions, Rayleigh test). Care should be taken in interpreting this finding because the center of mass analysis that was used to assess place field shifts only accounts for shifts of the primary place field and does not reflect firing rate changes in regions outside of the primary place field. The boxes indicate where the stimulus was placed in respect to the open field arena. For b-c, the stimulus was presented on the left side of the open field arena, and for e-f, the stimulus was presented in the lower left of the open field arena. For a and d, the chamber was empty.

Supplementary Table 1: Bonferroni post hoc tests for Fig 2cd.
*p<0.05; **p<0.01; ***p<0.001; ****p<0.0001

Subregion	Comparison	P value
CA1	Context 5’ vs 15’	***
	Context 5’ vs 30’	n.s.
	Context 15’ vs 30’	****
	Familiar 5’ vs 15’	***
	Familiar 5’ vs 30	n.s.
	Familiar 15’ vs 30-	**
	Novel 5’ vs 15’	n.s. (0.0627)
	Novel 5’ vs 30’	n.s.
	Novel 15’ vs 30’	n.s. (0.0589)
CA2	Context 5’ vs 15’	n.s.
	Context 5’ vs 30’	***
	Context 15’ vs 30’	**
	Familiar 5’ vs 15’	n.s.
	Familiar 5’ vs 30’	*
	Familiar 15’ vs 30-	***
	Novel 5’ vs 15’	n.s.
	Novel 5’ vs 30’	n.s.
	Novel 15’ vs 30’	*

Supplementary Methods

Predator and Prey Behavioral Task for Immediate Early Gene Expression (Supplementary Fig. 7)

Rats were singly housed upon arrival in a room with negative airflow for 2 weeks while being extensively handled on multiple days of the week. The behavioral testing was done in the same room they were housed in and in their home cages. Rats were habituated to an egg carton filled with clean Diamond Soft bedding once a day for 3 consecutive days. During habituation, a fruit flavored sucrose treat was buried in the bedding to encourage exploration of the bedding and the previous days egg carton (if any) was removed. On the fourth day (test day) animals were randomly assigned into three groups (N=5 per group): Control, Prey (mouse), and Predator (fisher cat). Power analyses were done a priori to determine animal numbers per group (80-90% statistical power using ANOVA followed by one sided t tests at the 0.05 level of significance comparing pairs of groups. Depending on the group, rats were presented an egg carton filled with Diamond Soft bedding adsorbed with nothing (Control), mouse bedding (Mouse), or Fisher Cat urine (PredatorPee.com). After ten minutes, the egg carton was removed and at a total time of 15 minutes (maximal nuclear *Arc* expression) the rat was sacrificed in an adjoining room by rapid decapitation without anesthesia. Brains were removed and flash frozen in isopentane cooled to -20°C in a dry ice/ethanol bath. Triple label *in situ* hybridization for *Arc*, *Egr1* and *Pcp4* was performed using RNAscope as detailed in the methods. Image acquisition and quantification were performed as described in the methods for the time course experiment except that only *Arc* foci were counted.

Supplementary References

1. Lu, L., Igarashi, K. M., Witter, M. P., Moser, E. I. & Moser, M.-B. Topography of Place Maps along the CA3-to-CA2 Axis of the Hippocampus. *Neuron* **87,** 1078–1092 (2015).
2. Mankin, E. A., Diehl, G. W., Sparks, F. T., Leutgeb, S. & Leutgeb, J. K. Hippocampal CA2 Activity Patterns Change over Time to a Larger Extent than between Spatial Contexts. *Neuron* **85,** 190–201 (2015).
3. Wersinger, S. R., Ginns, E. I., O'Carroll, A.-M., Lolait, S. J. & Young, W. S., III. Vasopressin V1b receptor knockout reduces aggressive behavior in male mice. *Mol. Psychiatry* **7,** 975–984 (2002).
